# Hydroxysafflor Yellow A Blocks HIF-1*α* Induction of NOX2 and Protects ZO-1 Protein in Cerebral Microvascular Endothelium

**DOI:** 10.3390/antiox11040728

**Published:** 2022-04-07

**Authors:** Yi Li, Xiao-Tian Liu, Pei-Lin Zhang, Yu-Chen Li, Meng-Ru Sun, Yi-Tao Wang, Sheng-Peng Wang, Hua Yang, Bao-Lin Liu, Mei Wang, Wen Gao, Ping Li

**Affiliations:** 1 State Key Laboratory of Natural Medicines, School of Traditional Chinese Pharmacy, China Pharmaceutical University, No. 24 Tongjia Lane, Nanjing 210009, China; 1620184494@cpu.edu.cn (Y.L.); 3221020587@stu.cpu.edu.cn (X.-T.L.); 3220020273@stu.cpu.edu.cn (P.-L.Z.); 3219020396@stu.cpu.edu.cn (Y.-C.L.); 3220020408@stu.cpu.edu.cn (M.-R.S.); yanghua@cpu.edu.cn (H.Y.); liubaolin@cpu.edu.cn (B.-L.L.); 2 State Key Laboratory of Quality Research in Chinese Medicine, Institute of Chinese Medical Sciences, University of Macau, Macau 999078, China; ytwang@um.edu.mo (Y.-T.W.); swang@um.edu.mo (S.-P.W.); 3 Leiden University-European Center for Chinese Medicine and Natural Compounds, Institute of Biology/SU BioMedicine, Leiden University, Sylviusweg 72, 2333 BE Leiden, The Netherlands; m.wang@biology.leidenuniv.nl

**Keywords:** hydroxysafflor yellow A, HIF-1*α*, NOX2, ZO-1, cerebral microvascular endothelium

## Abstract

Zonula occludens-1 (ZO-1) is a tight junction protein in the cerebrovascular endothelium, responsible for blood–brain barrier function. Hydroxysafflor yellow A (HSYA) is a major ingredient of safflower (*Carthamus tinctorius* L.) with antioxidative activity. This study investigated whether HSYA protected ZO-1 by targeting ROS-generating NADPH oxidases (NOXs). HSYA administration reduced cerebral vascular leakage with ZO-1 protection in mice after photothrombotic stroke, largely due to suppression of ROS-associated inflammation. In LPS-stimulated brain microvascular endothelial cells, HSYA increased the ratio of NAD^+^/NADH to restore Sirt1 induction, which bound to Von Hippel–Lindau to promote HIF-1*α*degradation. NOX2 was the predominant isoform of NOXs in endothelial cells and HIF-1*α* transcriptionally upregulated p47phox and Nox2 subunits for the assembly of the NOX2 complex, but the signaling cascades were blocked by HSYA via HIF-1*α* inactivation. When oxidate stress impaired ZO-1 protein, HSYA attenuated carbonyl modification and prevented ZO-1 protein from 20S proteasomal degradation, eventually protecting endothelial integrity. In microvascular ZO-1 deficient mice, we further confirmed that HSYA protected cerebrovascular integrity and attenuated ischemic injury in a manner that was dependent on ZO-1 protection. HSYA blocked HIF-1*α*/NOX2 signaling cascades to protect ZO-1 stability, suggestive of a potential therapeutic strategy against ischemic brain injury.

## 1. Introduction

The blood–brain barrier (BBB) is a continuous endothelial membrane that insulates the central nervous system from the peripheral immune system to maintain the functional homeostasis of the brain. In response to ischemic stroke, circulating endotoxin levels rise to aggravate brain injury, largely due to the impairment of endothelium integrity [1,2]. It is well established that dysregulated systemic immune and inflammatory responses participate in the destruction of the BBB and brain parenchymal damage [3].

Tight junction (TJ) is a specialized structure that is widely existing in both epithelial cells and endothelial cells, regarded as an important component of BBB. TJ is responsible for restricting the exchange of paracellular and intramembranous components (barrier function), as well as maintaining cell polarity (fence function) [4]. Zonula occludens-1 (ZO-1) is a scaffold protein that is the first identified as a cytosolic protein in the TJ. By binding to transmembrane proteins, ZO-1 drives the formation of TJ and integrates various signaling pathways, thereby establishing communication between the endothelium and brain [4]. The loss of ZO-1 and destroyed permeability of the BBB were observed in cerebral ischemic cases associated with oxidative stress and inflammatory responses [5]. LPS challenge is shown to impair ZO-1 protein in cerebral microvascular endothelial cells [6]. Inflammation and oxidative stress impair endothelial integrity, but the direct impact on TJ proteins is still unclear.

Oxidative stress is associated with vascular diseases and NADPH oxidases (NOXs) drive reactive oxygen species (ROS) production in the context of inflammation. Different NOX isoforms present in the blood vessel endothelium as the underlying causes of oxidative stress in various heart and cerebrovascular diseases [7]. NOXs consistently produce low levels of ROS to regulate endothelium-dependent relaxation and redox signaling under physiological conditions, but aberrant function produces excessive ROS leading to vascular injury. Nox2-derived ROS induces endothelial dysfunction associated with alternations in cerebral blood flow [8]. More pathological factors are involved in brain ischemic injury, but the cause of NOXs activation is remained to be identified. Recently, emerging evidence indicates the potential relation between hypoxia-inducible transcription factor-1*α* (HIF-1*α*) and NOXs. In colon cancer cells, inhibition of NOX1 expression led to a decrease in the expression of HIF-1*α* [9]. Furthermore, intermittent hypoxia also increased NOX2 expression in the brain cortex of mice, and this effect was abolished in HIF-1*α*^+/−^ mice [10]. HIF-1*α* is a transcriptional regulator sensitive to low oxygen tension, but its role in NOXs activation is remained to be elucidated.

Safflower (*Carthamus tinctorius* L.) is a kind of traditional Chinese medicine, and its preparation is widely used in the treatment of cardiovascular and cerebrovascular diseases. Hydroxysafflor yellow A (HSYA) is a major active ingredient of safflower, pharmacological studies have confirmed that HSYA improved myocardial ischemia-reperfusion injury through the suppression of NLRP3 inflammasome pathway and TLR4 signaling [11,12]. HSYA also inhibited neuronal apoptosis via inhibiting the p38 MAPK signaling pathway [13]. These events indicate the inhibitory effects of HSYA on inflammation. Furthermore, HSYA is widely used as an antioxidant because it possesses several phenolic hydroxyl groups that donate active hydrogen atoms to interdict oxidative damage (the structure of HSYA was shown in Figure S1). As medical preparations containing HSYA have been prescribed for the treatment of cardiocerebrovascular diseases, it is necessary to find an integrated mechanism for the better design of therapeutic strategy. In the present study, we identified that inflammation and disturbed redox status induced HIF-1α activation, which drove NOX2 induction in the cerebral microvascular endothelium. HSYA inhibited HIF-1α/NOX2 signaling cascades to protect ZO-1 from proteasomal degradation, resultantly protecting cerebral vessel integrity to attenuate brain injury. Given that conventional antioxidants display poor reactivity with endogenous ROS [7], targeting ROS-generating enzymes should be a more effective strategy for combating oxidative stress than scavenging highly reactive molecules.

## 2. Materials and Methods

### 2.1. Materials and Reagents

HSYA (purity ≥98%) was obtained from Chengdu Must Biotechnology Co, Ltd. (Chengdu, China). LPS (L2880), tert-butyl hydroperoxide (t-BHP, 416665), diamide (D3648), *N*-acetyl-l-cysteine (NAC, A9165), *β*-nicotinamide mononucleotide (NMN, N3501), rose bengal (330000), 2,3,5-triphenyltetrazolium chloride (TTC, T8877) and Evans blue (E2129) were purchased from Sigma (St. Louis, MO, USA). PX-478(HY-10231), cycloheximide (HY-12320), and 4-hydroxynonenal (4-HNE, HY-113466) were provided by Med Chem Express (Brea, CA, USA). gp91-ds-tat and sc gp91-ds-tat were obtained from GenScript Co., Ltd. (Nanjing, China).

### 2.2. Animals and Treatments

The study was approved by the Animal Ethics Committee of China Pharmaceutical University (protocol code: 2020-05-007 and date of approval: 14 May 2020). Male C57BL/6J mice (6–8 weeks old) were purchased from the Experimental Animal Center of Yangzhou University (Yangzhou, China). Mice were housed five per cage with a constant temperature of (24 ± 2 °C) for a 12:12 h light–dark cycle and given free access to standard food and water. They adapted to these conditions for 7 days before being used in the experiments.

For the preparation of the photothrombotic stroke model, male C57BL/6J mice were anesthetized and placed in a stereotaxic apparatus (Stoelting, Wood Dale, IL, USA). The skull was exposed after incising the midline and removing the periosteum, and then a cold light source (11,500 lux) converged by a 2 mm diameter fiber optic bundle was placed in the position of 1.5 mm to the right of the bregma. After injection of rose bengal (100 mg/kg, i.p.) for 5 min, the indicated position of the skull was illuminated for 15 min. Mice in the sham group underwent the same surgical procedures but received 0.9% saline instead of rose bengal. Following the illumination, the incision was sutured and the mice were transferred for postoperative rehabilitation.

The mice were randomly divided into 4 groups, with 15 mice in each group. After a photothrombotic stroke, mice were intraperitoneally injected with HSYA (50 mg/kg) and NAC (100 mg/kg) at the doses previously published [12,14]. The sham and the model group were given the same volume of normal saline. It was given once a day for 3 days. After administration, the spleen, liver, and kidney of the mice were weighed, and the length of the tibia was measured. The ratios of spleen, liver, and kidney weights to tibia length were calculated. At the same time, 5 mice from each group were randomly selected for TTC staining, Evans blue staining, and immunofluorescence staining of brain tissue.

For TTC staining, the brain of mice was dissected and frozen for 15 min. Afterward, centering on the largest diameter of the infarct area, the brain tissue was cut into coronal slices toward the head, respectively. The slices were stained with TTC (2%, *w*/*v*) for 10 min in a 37 °C water bath in the dark and fixed with 4% paraformaldehyde. After 24 h, brain slices were photographed and the infarct area was counted with ImageJ (the cerebral infarction rate = infarct area/total section area × 100%).

### 2.3. Hematoxylin and Eosin (HE) Staining

The spleen, liver, and kidney tissues were washed with PBS, fixed with 10% formaldehyde, dehydrated, embedded in paraffin, cut into 5 μm sections, and stained with hematoxylin and eosin. The tissue sections were observed under an inverted microscope (OLYMPUS, IXplore Standard, Tokyo, Japan).

### 2.4. Evans Blue Staining

After the administration, mice were injected with 2% Evans blue solution (4 mL/kg body weight) via the tail vein. Two hours after circulation, the mice were perfused with normal saline and paraformaldehyde, in turn, to wash out the residual Evans blue in the blood vessels and fix the tissue. Brain tissues were harvested and photographed, the infarct area was taken and homogenized with 50% trichloroacetic acid. After incubating overnight at 37 °C, tissues were centrifuged at 12,000× *g* for 20 min. The supernatant was collected and measured absorbance at 620 nm. The content of Evans blue leakage was quantitatively analyzed from the standard curve.

### 2.5. Cell Culture

bEnd.3 cells from iCell Bioscience Inc (Shanghai, China) were grown in DMEM (KeyGEN BioTECH, Nanjing, China) supplemented with 10% (*v*/*v*) FBS (Gibco, New York, NY, USA) at 37 °C in a humidified atmosphere of 5% CO_2_ in the air.

### 2.6. RNA Interference

To specifically knockdown Nox1, Nox2, ZO-1, and 20S proteasome, *Nox1* small interfering RNA (siRNA), *cybb* siRNA, *Tjp1* siRNA, *Psmb9* siRNA, or negative control (NC) siRNA were transfected into bEnd.3 with Lipofectamine™ 3000 transfection reagent (Invitrogen™, L3000015, Carlsbad, CA, USA) when cells grew to a confluence of 70–80%. The siRNA sequences were as follows: si*Nox1*: 5′- CGUCAGCUAUGGAGUUUAU -3′, si*Cybb*: 5′- CACCAUCUCUUUGUGAUCU-3′, si*Tjp1*: 5′-GCGACUAGCUGGUGGAAAU-3′, si*Psmb9*: 5′-GACUUGUUAGCGCA

UCUCAUA-3′, siNC: 5′-UUCUCCGAACGUGUCACGU-3′ (Shanghai GenePharma Co., Ltd., Shanghai, China). To carry out HIF-1*α* overexpression, bEnd.3 cells were transfected with pcDNA3.1-M_*Hif1α* and pcDNA3.1-M_NC plasmids. After culturing for 48 h, bEnd.3 cells were treated with indicated reagents for 16 h.

### 2.7. Measurement of Lactate Concentration and ELISA Assay

Blood samples collected from the experimental animals were centrifuged at 1000× *g* for 15 min to obtain serum. IL-1*β*, LPS, TNF-*α* in serum were measured by mouse IL-1*β* ELISA Kit (Neobioscience, Shenzhen, China), mouse LPS ELISA Kit (CUSABIO, Wuhan, China), and mouse TNF-*α* ELISA Kit (Neobioscience, Shenzhen, China), respectively.

bEnd.3 cells were treated with HSYA (10 μM) or NAC (2 mM) in the presence of LPS (100 ng/mL) for 16 h, referring to the dose in the published literature [11,13]. The cell supernatant was collected and lactate concentration was measured according to the manufacturer’s instructions (Jiancheng, A019-2-1, Nanjing, China). Additionally, the treated bEnd.3 cells were washed with PBS and dissociated with trypsin, then the freeze–thaw process was repeated to lyse cells. After centrifuging at 1500× *g* for 10 min at 4 °C, the supernatant was collected for the measurement of 4-HNE according to the manufacturer’s protocol of the 4-HNE ELISA Kit (Elabscience, Wuhan, China).

### 2.8. The Ratio of NAD^+^/NADH, GSH/GSSG, and the Content of MDA, Protein Carbonyl

bEnd.3 cells were treated with HSYA (10 μM) or NAC (2 mM) in the presence of LPS (100 ng/mL) for 16 h. NAD^+^/NADH ratio was measured according to the manufacturer’s protocol of NAD^+^/NADH Quantification Kit (Sigma, MAK037, St. Louis, USA). Briefly, after washing with cold PBS, bEnd.3 were collected, extracted with 400 µL NAD^+^/NADH extraction buffer, and centrifuged at 13,000× *g* for 10 min to remove insoluble material. Subsequently, half of the supernatant was collected for the detection of total NADH and NAD (NAD_total_), and the remaining half was incubated at 60 °C for 30 min to decompose NAD for the determination of NADH. Then, 10 µL NADH Developer was added for coloration, and the absorbance was measured at 450 nm. NAD^+^/NADH ratio was obtained according to the standard curve and the formula ratio = (NAD_total_-NADH)/NADH.

GSH/GSSG ratio was measured using GSH/GSSG Ratio Detection Assay Kit (Abcam, ab138881, Cambridge, UK). In brief, the treated bEnd.3 cells were washed with PBS and resuspended in Lysis Buffer. The supernatant was collected after centrifuging at 12000× *g* for 15 min at 4 °C. Deproteinization was carried out with trichloroacetic acid, which was neutralized to pH 4-6 with NaHCO_3_ later. Finally, fluorescence was measured by a multimode microplate reader (BERTHOLD Technologies, Bad Wildbad, Germany) at excitation of 490 nm and emission of 520 nm.

For measurement of MDA and protein carbonyl, the cell lysis buffer was centrifuged and detected according to the protocols of the manufacturer (Jiancheng, A003-4-1, A087-1-2, Nanjing, China). The absorbance was measured at 370 nm and 530 nm, to calculate the content of MDA and protein carbonyl, respectively.

### 2.9. Measurement of ROS and Lipid Peroxidation

For the measurement of intracellular ROS generation, the treated bEnd.3 cells were incubated with 5 μM dihydroethidium (DHE, Beyotime, S0063, Shanghai, China) at 37 °C for 30 min in the darkness. After washing with PBS 3 times, the intensity of cytosolic ROS was measured by a confocal laser-scanning microscope (Zeiss, LSM 800, Jena, Germany). For lipid peroxidation level, the treated bEnd.3 cells were incubated with a 10 µM Image-iT Lipid Peroxidation Sensor (Invitrogen, C10445, Carlsbad, CA, USA) for 30 min at 37 °C. Additionally, then nuclear staining was performed using Hoechst (Invitrogen, Carlsbad, CA, USA). Fluorescence images were captured under a confocal laser-scanning microscope (Zeiss, LSM 800, Jena, Germany) at emission 510/590 nm.

To observe ROS production in brain tissues, the brains of experimental animals were frozen and cut into slices. Then, the sections were incubated with 10 μM DCFH-DA (Beyotime, S0033S, Shanghai, China) for 30 min and DAPI for 10 min. The intensity of ROS was measured by a confocal laser-scanning microscope (Zeiss, LSM 800, Jena, Germany).

### 2.10. Immunofluorescence Staining

To detect the protein expression levels of CD31, Iba1, and ZO-1 in the brains of mice, the brain tissues were embedded in Tissue-Tek O.C.T. Compound (Sakura Finetek, Tokyo, Japan) and then cut into 5 μm thick slides. After fixation with 4% paraformaldehyde, the slides were permeabilized with 0.3% Triton X-100 and blocked with goat serum, followed by incubated overnight at 4 °C with primary antibodies (anti-CD31, 1 μg/mL, Abcam Cat# ab9498, RRID: AB_307284; anti-Iba1, 1:100, Abcam Cat# ab178847, RRID: AB_2832244; anti-ZO-1, 1 μg/mL, Abcam Cat# ab216880). After washing for three times, the slides were incubated with second antibody (Goat Anti-Mouse IgG H&L (Alexa Fluor^®^ 647), 1:500, Abcam Cat# ab150115, RRID: AB_2687948; Goat Anti-Rabbit IgG H&L (Alexa Fluor^®^ 488), 1:500, Abcam Cat# ab150077, RRID: AB_2630356; Donkey Anti-Rabbit IgG H&L (Alexa Fluor^®^ 594), 1:500, Abcam Cat# ab150076, RRID: AB_2782993) at room temperature for 2 h. Nuclei were stained with DAPI (1:1000, Bioworld Technology, BD5010, St. Paul, MN, USA) and an anti-fluorescence quenching agent (Beyotime, P0126, Shanghai, China) was added. Slides were observed and captured under a confocal laser-scanning microscope (Zeiss, LSM 800, Jena, Germany).

To detect the cellular immunofluorescence, the treated bEnd.3 cells were fixed with 4% paraformaldehyde, permeabilized with 0.1% Triton X-100 and blocked with 5% BSA. After that, cells were incubated with anti-HIF-1*α* (1:800, Cell Signaling Technology Cat# 36169, RRID: AB_2799095), anti-IgG (1:500, Beyotime Cat# A7016), anti-histone 3 (1:500, Cell Signaling Technology Cat# 4499, RRID:AB_10544537), anti-claudin 5 (1:100, Bioworld Technology Cat# BS1069, RRID: AB_1664057), anti-occludin (1:100, Bioworld Technology Cat# BS72035) or anti-ZO-1 (1 μg/mL, Abcam Cat# ab216880) overnight at 4 °C and goat anti-rabbit IgG H&L (Alexa Fluor^®^ 488) (1:500, Abcam Cat# ab150077, RRID: AB_2630356) at room temperature for 2 h. To achieve the co-localization of p47phox and Nox2, bEnd.3 cells were co-incubated with anti-p47phox (1:100, Santa Cruz Biotechnology Cat# sc-17845, RRID: AB_627986) and anti-Nox2 (1:100, Abcam Cat# ab80508, RRID: AB_1603890) at 4 °C overnight, followed by Goat Anti-Rabbit IgG H&L (Alexa Fluor^®^ 647) (1:500, Abcam Cat# ab150083, RRID: AB_2714032) and Goat Anti-Mouse IgG H&L (Alexa Fluor^®^ 488) (1:500, Abcam Cat# ab150113, RRID: AB_2576208) at room temperature for 2 h. Nuclei were located with DAPI (1:1000, Bioworld Technology, BD5010, St. Paul, MN, USA). Images were captured and analyzed under a confocal laser-scanning microscope (Zeiss, LSM 800, Jena, Germany).

### 2.11. Western Blotting and Immunoprecipitation

bEnd.3 cells were lysed with RIPA lysis buffer containing phosphatase and protease inhibitors (Roche, Basel, Switzerland) and the protein was quantified with a BCA protein assay kit (Thermo Scientific, 23225, Waltham, MA, USA). An equal amount of sample was separated with SDS-PAGE and then transferred to nitrocellulose membranes. The membranes were blocked in 5% non-fat milk for 2 h and incubated with primary antibodies (anti-Sirt1, 1:1000, Cell Signaling Technology Cat# 9475, RRID: AB_2617130; anti-Von Hippel–Lindau (VHL), 0.03 µg/mL, Abcam Cat# ab77262, RRID: AB_1524559; anti-HIF-1*α*, 1:1000, Cell Signaling Technology Cat# 36169, RRID: AB_2799095; anti-ZO-1, 1:1000, Abcam Cat# ab216880; anti-claudin 5, 1:1000, Bioworld Technology Cat# BS1069, RRID: AB_1664057; anti-occludin, 1:1000, Bioworld Technology Cat# BS72035; anti-p47phox, 1:500, Santa Cruz Biotechnology Cat# sc-17845, RRID: AB_627986; anti-acetylated-lysine, 1:1000, Cell Signaling Technology Cat# 9441, RRID: AB_331805; anti-proteasome 20S LMP2, 1:10,000, Abcam Cat# ab184172; anti-(Na+, K+)ATPase, 1:1000, Bioworld Technology Cat# BS90909; anti-*β*-actin, 1:2000, Proteintech Cat# 20536-1-AP, RRID: AB_10700003) at 4 °C overnight. Subsequently, strips were washed with TBST, and co-incubated with corresponding secondary antibodies (Goat Anti-Rabbit IgG (H+L)-HRP, 1:5000, Bioworld Technology Cat# BS13278, RRID: AB_2773728; Rabbit Anti-Goat IgG (H+L)-HRP, 1:5000, Bioworld Technology Cat# BS30503; Goat Anti-Mouse IgG (H + L) HRP, 1:10,000, Bioworld Technology Cat# BS12478, RRID: AB_2773727) at room temperature for 2 h. After further washing, the immunoreactive bands were developed by an ECL kit and then analyzed by Image-Pro Plus 6.0 software.

For immunoprecipitation assay, the lysate was collected and incubated with antibody (anti-Sirt1, 1:30, Abcam Cat# ab189494, RRID: AB_2864311; anti-Nox2, 1:50, Abcam Cat# ab80508, RRID: AB_1603890; anti-VHL, 1:50, Abcam Cat# ab77262, RRID: AB_1524559; anti-ZO-1, 1:100, Abcam Cat# ab216880) overnight at 4 °C and then combined with protein A + G agarose beads (Med Chem Express, HY-K0202, Brea, CA, USA). The mixture was shaken at 4 °C for 4 h to fully combine. The beads were washed with PBS 5 times and boiled with 1% SDS loading buffer for Western blot assay.

Carbonyl introduced into proteins by oxidative reactions was detected with Protein Carbonyl Assay Kit (Western Blot) (Abcam, ab178020, Cambridge, UK). bEnd.3 cells were solubilized with 1× extraction buffer and incubated on ice for 20 min. Protein in cells was treated according to the manufacturer’s protocol. Then, Western blotting was performed to characterize the level of protein carbonyl.

### 2.12. Quantitative Real-Time PCR (qRT-PCR)

Total RNA from bEnd.3 cells were isolated with an RNA isolator (Vazyme, Nanjing, China). The concentration of RNA was measured by a NANO-100 micro-spectrophotometer (ALLSHENG, China), and the absorbance at 260/280 nm was considered to detect the purity of RNA. HiScript^®^ Q RT SuperMix for qPCR (Vazyme, Nanjing, China) was used to synthesize cDNA from RNA. Subsequently, qRT-PCR was performed with ChamQ SYBR Color qPCR Master Mix (Vazyme, Nanjing, China) on LightCycler 480 II (Roche, Basel, Switzerland). mRNAs in the tested samples were standardized with *Actb*, and the 2^−^^ΔΔCt^ method was used for relative quantification. Primer sequences are listed in Table S1.

### 2.13. Dual-Luciferase Reporter Gene Assay

The Mouse_*Ncf1* promoter (−2142 to −1) and Mouse_*Cybb* promoter (−1880 to +74) were synthesized and inserted in the pGL3-basic vector between Mlul and Xhol sites (Genomeditech Co., Ltd., Shanghai, China). bEnd.3 were transfected with pEX3-*Ncf1*, pEX3-*Cybb*, pcDNA3.1-M_*Hif1α*, or pcDNA3.1-M_NC using Lipofectamine™ 3000 reagent and P3000™ Reagent. pRL-SV40 renilla luciferase expression plasmid, a normalized control, were co-transfected into bEnd.3 cells. After 24 h transfection and 16 h stimulation of indicated reagents, bEnd.3 cells were lysed using passive lysis buffer (Promega, E1941, Madison, WI, USA); then, the dual-luciferase assay system (Promega, E2920, Madison, WI, USA) was used to test the luciferase activities.

### 2.14. Chromatin Immunoprecipitation (ChIP) Analysis

To detect the connection of HIF-1*α* with p47phox and Nox2, target chromatin was extracted and precipitated following the manufacturer’s instructions from the SimpleChIP Enzymatic Chromatin IP Kit (Cell Signaling Technology, 9002, Danvers, MA, USA). Briefly, formaldehyde (sigma, 252549, St. Louis, MO, USA) was added to the treated bEnd.3 cells at a final concentration of 1% to make protein cross-linked to DNA. After 10 min, the crosslinking was terminated by the addition of glycine. Cells were washed twice with precooled PBS and collected with PBS buffer containing protease inhibitor cocktail. Chromatin was broken into 150–900 bp fragments by using micrococcal nuclease and sonication. Anti-HIF-1*α* was applied for immune precipitation of the target DNA-protein complexes, while normal Rabbit IgG (Cell Signaling Technology, 2729, Danvers, MA, USA) and H3 antibody (Cell Signaling Technology, 4620, Danvers, MA, USA) was used as a negative control and positive control, respectively. An equal part of each sample had been set aside as input control before antibody processing. The bound DNA fragments were eluted and purified and then amplified using loci-specific primer. The primer sequences are shown in Table S2. Final data were normalized with the Input control of the corresponding sample.

### 2.15. Adeno-Associated Virus Serotype 2 (AAV2)-Based Endotheliotropic-Specific Knockdown of Tjp1

AAV2/br1-TIE-mir30-m-*Tjp1* (NC_000073.7) and AAV2/br1-TIE-NC (negative control) viruses were designed by Hanbio Biotechnology (Shanghai, China). For the endotheliotropic-specific *Tjp1* knockdown, mice were injected with 150 μL of AAV2/br1-TIE-mir30-m-*Tjp1* at a concentration of 1.5 × 10^12^ vg/mL viral genomes or AAV2/br1-TIE-NC through the caudal vein. The target sequence of AAV2/br1-TIE-mir30-m-*Tjp1* is 5′-GCGACTAGCTGGTGGAAAT-3′, and the sequence of AAV2/br1-TIE-NC is 5′-TTCTCCGAACGTGTCACGT-3′. Three weeks later, the knockout efficiency of ZO-1 was confirmed. Next, the mice were performed with a photothrombotic stroke model and given HSYA (50 mg/kg) for three consecutive days. Mice in the sham group and the model group were given the same volume of normal saline. There were 10 mice in each group, after administration, 5 mice from each group were randomly selected for Evans blue staining to evaluate the BBB function of the knockout mice and immunofluorescence staining in the peri-infarct zones of the brain tissue.

### 2.16. Transendothelial Electrical Resistance (TEER) Value and FITC–Dextran Paracellular Permeability Determination

TEER value is generally used to evaluate the integrity of bEnd.3 monolayer cells and the permeability of BBB in vitro. bEnd.3 cells were suspended and seeded in the apical chamber of 12-well Transwell inserts (3460, CORNING, New York, NY, USA) at a volume of 600 μL/well, while 1.5 mL of complete medium was added in the basolateral chamber. After 7 d culture in a sterile incubator, bEnd.3 cells were treated with LPS (100 ng/mL), HSYA (10 μM), and NAC (2 mM) for 16 h. After washing the chambers with HBSS, the TEER value was measured by the R/V Meter of Epithelium (RE1600, Beijing KingTech Technology Co. Ltd., Beijing, China). The TEER values were calculated by subtracting the resistance of a cell-free insert from an insert with cells and by subsequent multiplying by the total membrane surface area to obtain the resistance value in Ω•cm^2^.

After the measurement of the TEER value, 0.5 mL of DMEM containing 1 mg/mL FITC–Dextran (70 kDa, SIGMA, St. Louis, MO, USA) was added to the apical chamber. Following 1 h incubation in the dark, the fluorescence intensity of FITC–Dextran in the basolateral and apical was measured by a multimode microplate reader (BERTHOLD Technologies, Bad Wildbad, Germany) at excitation 485 nm and emission 525 nm. The equation P_dextran_ = (RFU_basolateral_/RFU_apical_) (V) (1/time) (1/area) was used to calculate the permeability coefficient, and permeability fold changes were calculated between control and treatment conditions for each experiment.

### 2.17. Statistical Analysis

All statistical tests are performed by using GraphPad Prism 8.4.2, and the data are expressed as the means ± SD (*n* ≥ 5). Statistical significance is determined by analysis of ordinary one-way ANOVA, followed by Tukey’s test. All experiments were randomized and blinded to avoid unintentional bias and to generate groups of equal size; values of *p* < 0.05 were considered statistically significant.

## 3. Results

### 3.1. HSYA Protected Brain Microvessels against Ischemic Injury

**Figure 1:**
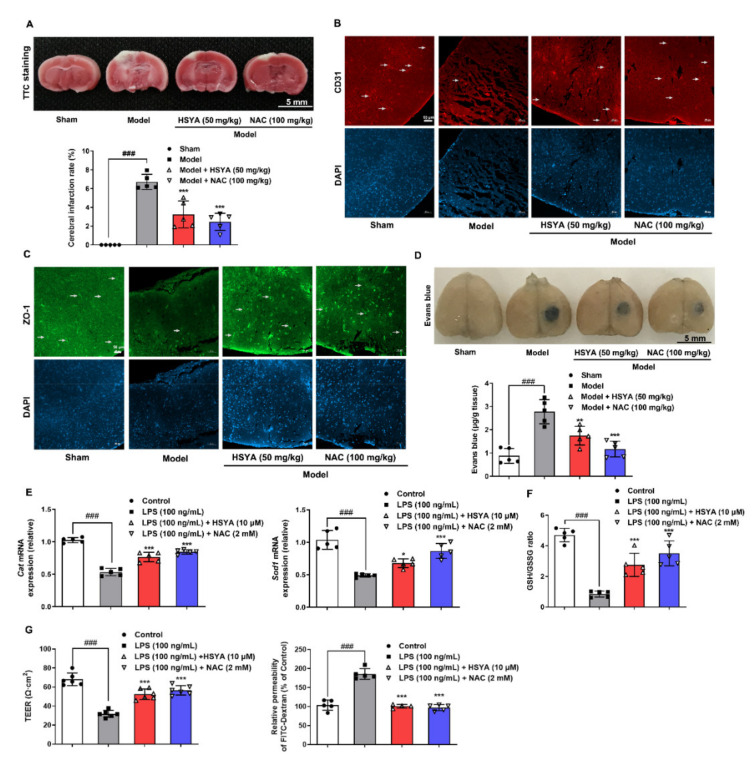
HSYA protected brain microvessels against ischemic injury. Mice were intraperitoneally injected with hydroxysafflor yellow A (HSYA) and NAC for 3 days after photothrombotic stroke: (**A**) the brain tissue coronal sections were stained with TTC staining, and the infarct area was counted with Image J; (**B**,**C**) CD31 and zonula occludens-1 (ZO-1) expressions were detected by immunofluorescence in the peri-infarct zones of brain tissue (scale bar: 50 μm); (**D**) mice were injected with 2% Evans blue via tail vein. Brain tissue was removed and photographed, and the amount of Evans blue leaking into the brain tissue was quantified; (**E**) the mRNA levels of catalase (*Cat*) and superoxide dismutase 1 (*Sod1*) were detected by qPCR; (**F**) the GSH/GSSG ratio was measured in the treated bEnd.3 cells; (**G**) the transendothelial electrical resistance (TEER) values and FITC-dextran paracellular permeability determination across the bEnd.3 monolayer cells. All data are presented as mean ± SD of five independent experiments. ^###^ *p* < 0.001 vs. indicated group, * *p* < 0.05, ** *p* < 0.01, *** *p* < 0.001 vs. LPS group.

As male mice were documented to be sensitive to brain ischemic injury [15], we prepared a photothrombotic stroke model in male C57BL/6J mice and HSYA (50 mg/kg) was administrated by intraperitoneal injection once a day for 3 consecutive days after surgery. The results of TTC staining showed that HSYA administration reduced brain infarct volume (Figure 1A). Since the loss of BBB integrity is the early event of ischemic cerebral injury, we examined the expression of platelet endothelial cell adhesion molecule 1 (PECAM1, also known as CD31) in the brain. Immunofluorescence staining revealed that HSYA prevented the loss of CD31 in the peri-infarct zones of the brain (Figure 1B). The intracellular scaffolding protein ZO-1 is the pivotal component of tight junctions in the BBB endothelial cells, and the loss of ZO-1 protein in the peri-infarct zones was also restored by HSYA (Figure 1C). Meanwhile, HSYA significantly reduced the Evans blue extravasation into the brain parenchyma (Figure 1D). ROS scavenger *N*-acetylcysteine (NAC) exhibited effects similar to HSYA, suggestive of the involvement of oxidative stress in BBB injury (Figure 1A–D). Meanwhile, HSYA and NAC also reduced microglia infiltration and ROS production in the peri-infarct zones of the brain, as expected (Figure S3A,B). No describable impacts on kidney, liver, and spleen were observed in mice after HSYA administration at the dose of 50 mg/kg (Figure S2A,B), indicative of the safety of HSYA administration at the given dose.

Stroke triggered peripheral inflammatory responses, indicated by the elevated levels of circulating IL-1*β* and TNF-*α*, which were lowered by HSYA and NAC (Figure S3C). Notably, abnormally increased LPS contents in the blood were also reduced by HSYA and NAC treatments, respectively (Figure S3C). As the main component of the BBB, brain microvascular endothelial cells and their surrounding tight junction proteins give full play to the characteristics of the blood–brain barrier to regulate the entry and exit of substances. bEnd.3 cells are derived from mouse cerebral microvascular endothelial cells with barrier properties, so we selected these cells for the subsequent experiments. Given the rise in circulating endotoxin after stroke, we stimulated cerebral microvascular endothelial cells (bEnd.3 cells) with LPS and found that HSYA suppressed gene expression of proinflammatory cytokines (Figure S3D). Next, we examined the potency of HSYA in the suppression of oxidative stress. HSYA increased mRNA levels of the antioxidant enzymes including catalase (*Cat*) and superoxide dismutase (*Sod1*) (Figure 1E) and raised the ratio of GSH/GSSG (Figure 1F), well demonstrating its ability to enhance antioxidative defense. Trans-endothelial electrical resistance (TEER) value and FITC–dextran permeability assay were used to evaluate endothelial integrity. bEnd.3 cells were seeded at a density of 10^5^ cells/cm^2^ in the apical chamber of 12-well Transwell inserts, and LPS treatment reduced the TEER value to 46.7%, which was restored to 77.1% by HSYA treatment. Meanwhile, HSYA treatment also effectively reduced the paracellular diffusion of FITC–dextran (70 kDa) across endothelial monolayer cells (Figure 1G). ROS scavenger NAC showed a similar regulation, suggesting that HSYA restrained oxidative stress to protect brain microvessels integrity.

### 3.2. HSYA Prevented HIF-1α Accumulation

**Figure 2:**
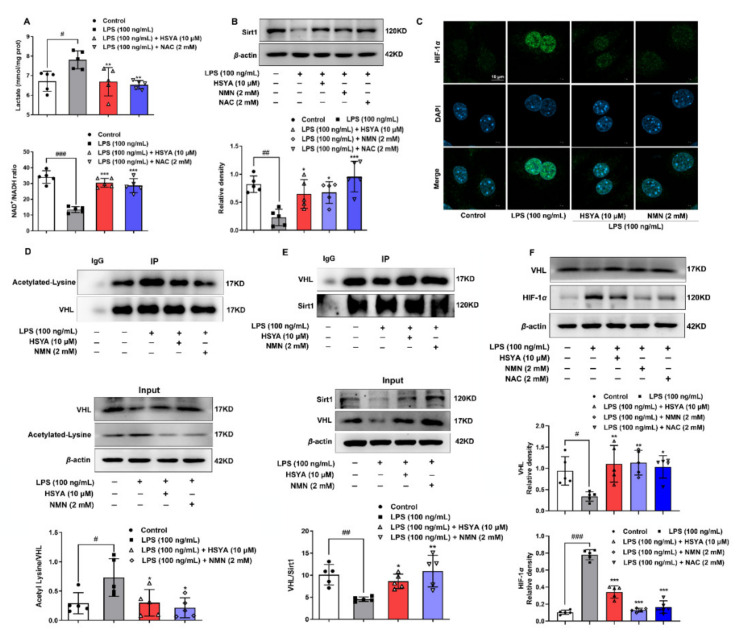
HSYA prevented HIF-1*α* activation: (**A**) lactate content and NAD^+^/NADH ratio were measured in bEnd.3 cells exposed to LPS insult; (**B**) protein expression of Sirt1; (**C**) the view of HIF-1*α* transport into the nucleus under a confocal microscope (scale bar: 10 μm); (**D**,**E**) the acetylation level of Von Hippel–Lindau (VHL) and the binding of Sirt1 to VHL were determined by immunoprecipitation; (**F**) VHL and HIF-1*α* expression in LPS-induced bEnd.3 cells. All data are presented as mean ± SD of five independent experiments. ^#^ *p* < 0.05, ^##^ *p* < 0.01, ^###^ *p* < 0.001 vs. indicated group, * *p* < 0.05, ** *p* < 0.01, *** *p* < 0.001 vs. LPS group.

Following ischemic insult, the level of ROS is dependent on the shifted NAD(H) redox status, while NAD^+^ is an essential co-factor for the regulation of cellular energy homeostasis and antioxidant defenses. Similar to metabolic reprogramming in macrophage activation [16], LPS shifted metabolism toward glycolysis in endothelial cells, evidenced by the elevated level of lactate with a corresponding decrease in the ratio of NAD^+^/NADH (Figure 2A). HSYA treatment reduced lactate production and increased the ratio of NAD^+^/NADH, and the action had a contribution to restoring Sirt1 protein expression because Sirt1 is an NAD^+^-dependent deacetylase (Figure 2B). In support, NAD^+^ precursor *β*-nicotinamide mononucleotide (NMN) increased Sirt1 protein abundance against LPS (Figure 2B). For the view of HIF-1*α* nuclear localization, we used IgG and histone 3 as the negative and positive control to exclude non-specific staining (Figure S4). Immunofluorescence staining showed that HSYA reduced HIF-1*α* nuclear localization responding to LPS insult (Figure 2C). NMN reduced nuclear HIF-1*α* expression, suggestive of inactivation of HIF-1*α* by Sirt1, as HIF-1*α* stability is sensitive to acetylation modification [17]. By recognizing the hydroxylated proline residue of HIF-1*α*, the VHL E3 ubiquitin ligase prevents HIF-1*α* accumulation via proteasomal degradation [18]. LPS insult increased VHL acetylation and blocked the binding of Sirt1 to VHL that were reversed by HSYA and NMN (Figure 2D,E). When protein synthesis was inhibited by cycloheximide, LPS stimulation promoted VHL protein degradation, and a significant effect was observed after 4 h incubation. In contrast, NMN repletion protected VHL protein abundance from degradation (Figure S5). The VHL E3 ubiquitin ligase complex can bind and catalyze the ubiquitination of HIF-1*α*, eventually leading to HIF-1*α* proteasomal degradation. As expected, HSYA and NMN increased VHL protein expression and reduced HIF-1*α* accumulation, respectively (Figure 2F). Similar regulation was also observed in NAC treatment, consistent with the fact that ROS inactivates VHL to promote HIF-1*α* accumulation [17]. These results indicated that HSYA prevented HIF-1*α* accumulation dependent on NAD^+^/Sirt1 cascades against oxidative stress.

### 3.3. HIF-1α Transcriptionally Regulated NOX2

**Figure 3:**
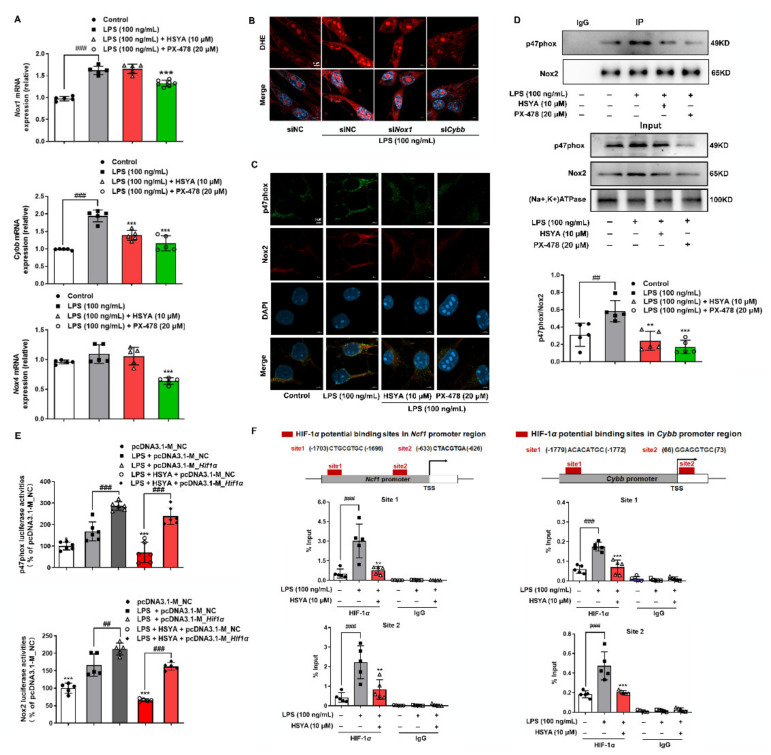
HIF-1*α* transcriptionally regulated NOXs: (**A**) gene expression of *Nox1*, *Cybb*, and *Nox4* in LPS-induced bEnd.3 cells; (**B**) O^2−^ production was viewed by dihydroethidium (DHE) staining in *Nox1* or *Cybb* silencing cells (scale bar: 5 μm); (**C**) immunofluorescence indicated the protein co-localization of p47phox (green) and Nox2 (red) in LPS-induced bEnd.3 cells (scale bar: 5 μm); (**D**) the binding of p47phox to Nox2 was determined by immunoprecipitation; (**E**) bEnd.3 cells were transfected with vectors encoding HIF-1*α* (pcDNA3.1-M_*Hif1α*), and the luciferase report activity of p47phox and Nox2 was measured by luciferase reporter gene kits; (**F**) ChIP-qPCR analysis of HIF-1*α* binding to the promoters of *Ncf1* and *Cybb* in bEnd.3 cells in response to LPS insult. All data are presented as mean ± SD of five independent experiments. ^##^ *p* < 0.001, ^###^ *p* < 0.001 vs. indicated group, ** *p* < 0.01, *** *p* < 0.001 vs. LPS group.

Each of the NOX isoforms comprises a core catalytic subunit with several regulatory subunits, and the assembly is needed for enzymatic activation. To address the role of HIF-1*α* in the regulation of NOXs in the cerebral microvascular endothelium, we examined NOX1, NOX2, and NOX4 induction, as NOX3 is mainly in fetal tissues of the kidney and liver and NOX5 is predominantly found in lymphocytes [19]. LPS stimulation increased gene expressions of *Nox1* and *Cybb* (encoding NOX2) in a manner dependent on HIF-1*α* because the gene induction was blocked by HIF-1*α* inhibitor PX-478, while *Nox4* induction was not affected (Figure 3A). Additionally, HSYA treatment lowered *Cybb* expression without influence on *Nox1* (Figure 3A). p47phox is a regulatory subunit of both NOX1 and NOX2, and its gene expression (*Ncf1* encoding p47phox) was also reduced by HSYA (Figure S6A). To distinguish the contribution of NOX1 and NOX2 to ROS generation, we used the small interference technique to silence their genes and found that intracellular ROS levels (indicated by DHE labeling) significantly decreased in *Nox2* silencing cells, compared with *Nox1* knockdown (Figure 3B and Figure S7A,B), indicating that NOX2 was the predominant isoform that promoted ROS production in response to LPS insult. As a specific inhibitor of Nox2, gp91 ds-tat significantly reduced ROS production in LPS-induced bEnd.3 cells (Figure S6B), providing evidence that Nox2 is the main source of ROS production in microvascular endothelial cells. The view of immunofluorescent staining showed that HSYA blocked cytosolic p47phox translocation to the membrane with the binding to Nox2 upon LPS stimulation (Figure 3C), and this role was further confirmed by the examination with immunoprecipitation (Figure 3D). Therefore, we concluded that HSYA combated inflammation-associated ROS production by suppressing NOX2 activation in endothelial cells. HIF-1*α* inhibitor PX-478 exhibited an inhibitory effect similar to HSYA, suggestive of the involvement of HIF-1*α* in NOX2 activation (Figure 3C,D). In line with this, overexpression of HIF-1*α* increased LPS-induced luciferase report activity of p47phox and Nox2 in bEnd.3 cells; HSYA treatment decreased *Ncf1* and *Cybb* promoter activity, but the action was abrogated by HIF-1*α* overexpression (Figure 3E). JASPAR database predicted two potential HIF-1*α*-binding sites in the p47phox promoter region. Indeed, HIF-1*α* promotes its target genes expression by binding to the hypoxia response element (HRE), with the core sequence 5′-RCGTG-3′, which is present in the p47phox promoter. ChIP-qPCR showed that LPS stimulation increased the interaction of HIF-1*α* with the *Ncf1* promoter at site 1 and site 2 and thus promoted p47phox expression, which was an inverse effect of HSYA treatment. Similarly, HSYA also lowered the LPS-increased binding of HIF-1*α* to *Cybb* promoter at site 1 and site 2 (Figure 3F). These results indicated that blocking HIF-1*α* transcriptional regulation was a way for HSYA to suppress NOX2 activation-derived ROS production.

### 3.4. HSYA Protected ZO-1 from Oxidative Damage

**Figure 4:**
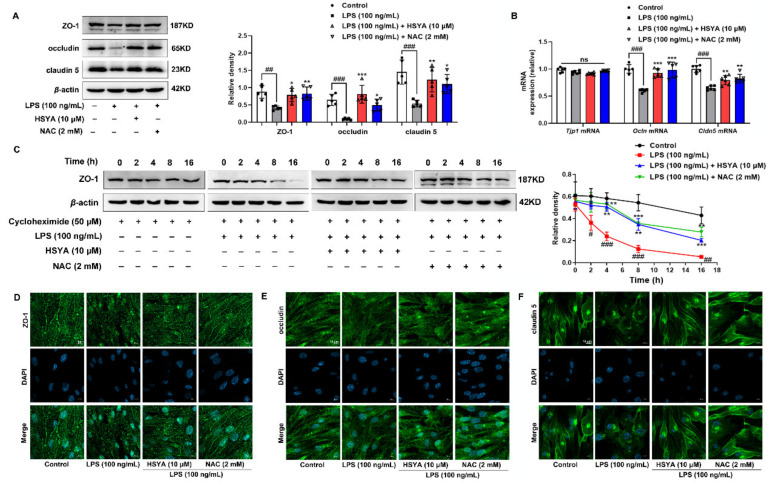
HSYA protected ZO-1 from oxidative damage: (**A**) protein expression of ZO-1, occludin, and claudin 5; (**B**) gene expression of ZO-1 (*Tjp1*), occludin (*Ocln*), and claudin 5 (*Cldn 5*); (**C**) ZO-1 protein degradation at the indicated time was determined when protein synthesis was inhibited by cycloheximide in LPS-stimulated bEnd.3 cells; (**D**–**F**) immunofluorescence showed the expression of ZO-1, occludin, and claudin 5 protein in bEnd.3 cells in the presence of LPS (scale bar: 10 μm). All data are presented as mean ± SD of five independent experiments. ^#^ *p* < 0.05, ^##^ *p* < 0.01, ^###^ *p* < 0.001 vs. indicated group, * *p* < 0.05, ** *p* < 0.01, *** *p* < 0.001 vs. LPS group; ns: no significant difference.

ZO-1 is localized at TJ sites with the connection to claudins and occludin, and its deficiency leads to TJ damage due to the lack of claudins polymerization and BBB breakdown [5]. LPS stimulation inhibited the protein expression of ZO-1, occludin, and claudin 5 in endothelial cells, which were reversed by HSYA treatment (Figure 4A). Interestingly, the mRNA expression of ZO-1 (*Tjp1*) revealed comparable values upon LPS stimulation, excluding potential transcriptional regulation (Figure 4B). Next, we incubated microvascular endothelial cells with cycloheximide to inhibit protein synthesis and found that LPS impaired the stability of ZO-1 protein, evidenced by continuous degradation from 2 to 16 h after treatment, whereas HSYA treatment substantially improved ZO-1 stability (Figure 4C). In line with these observations, immunofluorescence showed that ZO-1 distribution along the periphery of cells was disrupted after LPS stimulation, but the impairment was normalized by HSYA treatment (Figure 4D). Similar to the alterations in ZO-1 protein, HSYA also upregulated occludin and claudin 5 protein levels in LPS-stimulated endothelial cells (Figure 4E,F). NAC treatment maintained the stability of ZO-1 protein as well, rendering us to speculate that ZO-1 protein degradation should be a result of ROS damage. Similar to LPS insult, H_2_O_2_, and diamide impaired ZO-1 protein expression especially in the cell–cell contact site, but these effects were attenuated by HSYA treatment, providing evidence to support our speculation (Figure S8A,B).

### 3.5. HSYA Protected ZO-1 Protein Stability from 20S Proteasomal Degradation

**Figure 5:**
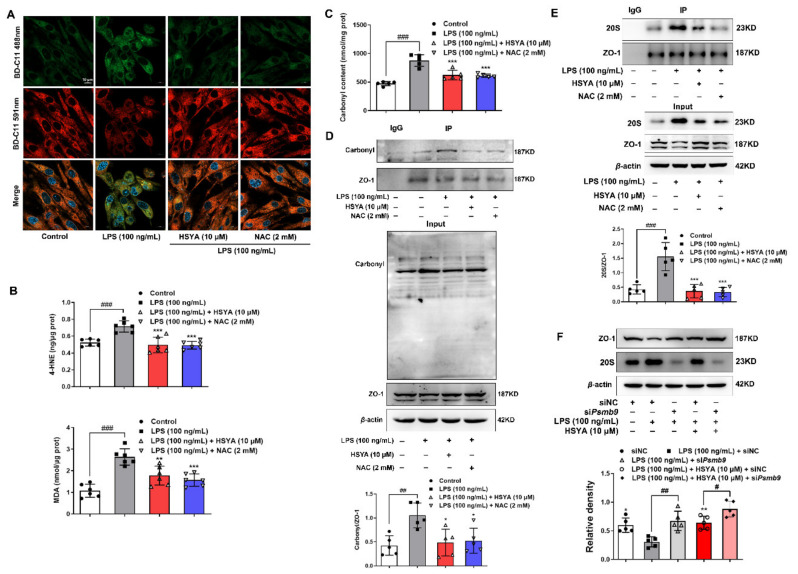
HSYA protected ZO-1 protein stability from 20S proteasome degradation: (**A**) the level of lipid peroxidation was observed, in which reducing lipid was in red and lipid peroxidation was in green (scale bar: 10 μm); (**B**,**C**) the concentration of 4-hydroxynonenal (4-HNE) and malondialdehyde (MDA) and the carbonyl content in LPS-induced bEnd.3 cells; (**D**) the carbonylation level of ZO-1 was determined by immunoprecipitation; (**E**) the immunoprecipitation assay of the binding of 20S to ZO-1 in bEnd.3 cells; (**F**) bEnd.3 cells were treated with HSYA in the presence of LPS when transfected with 20S siRNA (si*Psmb9*) or control siRNA (siNC). Protein expression of ZO-1 was determined (*n* = 5 in each group). All data are presented as mean ± SD of five independent experiments. ^#^ *p*< 0.05, ^##^ *p* < 0.01, ^###^ *p* < 0.001 vs. indicated group, * *p* < 0.05, ** *p* < 0.01, *** *p* < 0.001 vs. LPS group.

Oxidative stress induces lipid peroxidation, resulting in the generation of reactive lipid aldehydes including 4-HNE and malondialdehyde (MDA), which can destroy protein structure and stability. Therefore, we viewed lipid peroxidation using a lipid peroxidation sensor in LPS-stimulated bEnd.3 cells and found that HSYA and NAC treatment inhibited lipid peroxidation (Figure 5A) and reduced 4-HNE and MDA accumulation (Figure 5B). Lipid peroxidation products can covalently modify proteins to cause protein carbonylation. We next examined the influence of HSYA on carbonyl modification of ZO-1. Consistent with an increase in the total carbonyl contents (Figure 5C), ZO-1 carbonylation was strengthened in LPS-treated endothelial cells (Figure 5D). HSYA and NAC attenuated carbonyl modification of ZO-1 with downregulation of carbonyl level in bEnd.3 cells (Figure 5C,D). The 20S proteasome is responsible for the recognition and degradation of oxidized proteins [20]. For this, we examined the affinity of ZO-1 protein with 20S proteasome by immunoprecipitation in the setting of oxidative stress, and the data showed that HSYA and NAC treatments effectively blocked the binding of 20S proteasome to ZO-1 protein (Figure 5E). In line with this regulation, 20S proteasome knockdown reduced LPS-induced ZO-1 degradation and further potentiated the effect of HSYA on ZO-1 stabilization (Figure 5F and Figure S7C). We treated bEnd.3 cells with 4-HNE and found that it promoted the interaction of 20S proteasome to ZO-1 and decreased the stability of ZO-1 protein (Figure S8C), resultantly lowering the TEER value and raising the concentration of FITC–dextran (Figure S8D). Together, these results provided evidence that HSYA protected ZO-1 from 20S proteasomal degradation, contributing to protecting brain microvessels integrity.

### 3.6. ZO-1 Deficiency Attenuated the Protective Effects of HSYA during Cerebral Ischemic Injury

**Figure 6:**
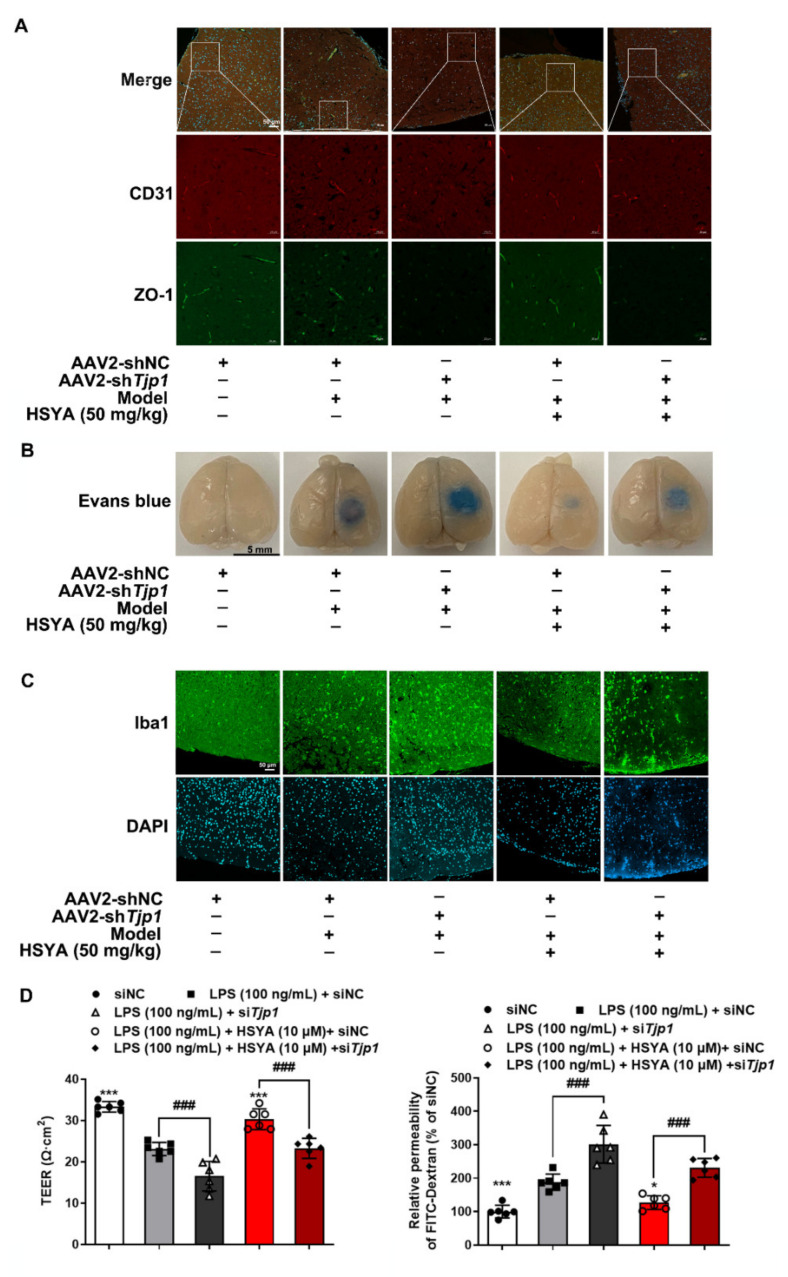
ZO-1 deficiency attenuated protective effects of HSYA during cerebral ischemic injury. Male C57BL/6J mice were injected with AAV2/br1-TIE-mir30-m-*Tjp1* and AAV2/br1-TIE-NC (negative control) viruses to build ZO-1 deficiency mice. Additionally, the mice were administrated with HSYA for 3 consecutive days: (**A**) immunofluorescence staining of ZO-1 protein expression in the peri-infarct zones of brain tissue (scale bar: 50 μm). Green: CD31, red: ZO-1; (**B**) Evans blue staining was used to observe the permeability of blood–brain barrier in mouse brain tissue. After injection of 2% Evans blue for 2 h, the brain tissue of the mice was removed and photographed; (**C**) immunofluorescence of Iba1 in the peri-infarct zones of brain tissue (scale bar: 500 μm); (**D**) bEnd.3 cells were transfected with siRNA against ZO-1 (si*Tjp1*) or control siRNA (siNC) and then treated with HSYA in the presence of LPS. TEER values, and the absorbance of FITC–dextran across the bEnd.3 monolayer cells were measured. All data are presented from five independent experiments. ^###^ *p* < 0.001 vs. indicated group, * *p* < 0.05, *** *p* < 0.001 vs. LPS group.

To further confirm the ZO-1-dependent role of HSYA in vivo, cerebral microvascular ZO-1 was knocked down by tail vein injection of AAV2/br1-TIE-mir30-m-*Tjp1* in mice. The protein expression of ZO-1 in the peri-infarct zones of the brain was markedly reduced, and the effect of HSYA was blocked by microvascular knockdown of *Tjp1,* which encodes ZO-1 protein (Figure 6A). Meanwhile, HSYA decreased the Evans blue extravasation in the ischemic brain in a manner that was dependent on ZO-1 protein (Figure 6B). The view of immunofluorescence showed that HSYA decreased the aggregation of microglia in the peri-infarct zones of the brain dependent on ZO-1 (Figure 6C). Moreover, ZO-1 knockdown diminished the protective effects of HSYA on endothelial integrity in LPS-induced bEnd.3 cells (Figure 6D and Figure S7D). Collectively, these results reproduced the in vitro findings and confirmed that HSYA preserved ZO-1 stability to protect brain microvessels integrity and BBB function.

## 4. Discussion and Conclusions

The tight junctions are essential for the integrity of the BBB, wherein ZO-1 stability is a key to the protection of endothelial function. Although there are different isoforms of NOXs in the cerebral microvessels, we identified NOX2 as the predominant isoform that was sensitive to HIF-1*α* activation. HSYA inhibited HIF-1*α*/NOX2 signaling cascades to protect ZO-1 from proteasomal degradation, addressing that protection of ZO-1 stability was a way for pharmacological intervention to ensure BBB integrity against ischemic injury.

Cerebral thrombosis is one of the most common types of cerebrovascular disease, and the main cause is atherosclerosis and intima injury, which causes local platelet aggregation and fibrin agglutination, leading to vessel occlusion and consequent ischemic brain injury. Brain injury causes a systemic stress response in the body and activates macrophages in adipose tissue and liver tissue to trigger peripheral inflammatory responses [21]. In patients with acute stroke, elevated levels of circulating inflammatory cytokines are positively correlated to stroke-associated damage [22]. We found that circulating levels of inflammatory cytokines including IL-1*β* and TNF-*α* were significantly upregulated in mice subjected to photothrombotic stroke. Interestingly, the concentration of LPS was also elevated in the periphery blood. By TLR4 activation, LPS evokes oxidative stress and impairs nitric oxide synthase (eNOS) to induce endothelial dysfunction [23]. It has been reported that BBB permeability and endothelial cell damage are more pronounced in LPS-stimulated brains, accompanied by monocyte infiltration, neuron death, and microglia aggregation [24]. Consistent with the role in the suppression of neuroinflammation [25], HSYA reduced proinflammatory cytokines production and combated oxidative stress, having a contribution to preventing the damage to cerebral microvessels.

NOXs also contribute to neuroinflammation and neuronal hyperexcitability in mice after sepsis [26]. NOXs are an important source of ROS in cardiovascular diseases [7]. In LPS-activated macrophages, succinate induces HIF-1*α* accumulation to evoke inflammation [27]; however, we found that in microvascular endothelial cells, HIF-1*α*-mediated NOXs activation was sensitive to altered metabolism and redox state. LPS shifted metabolism to glycolysis, which consumed NAD^+^ to inactivate Sirt1 because Sirt1 is an NAD^+^-dependent deacetylase. Sirt1 promotes HIF-1*α* degradation by VHL induction in skeletal muscle [17]. By raising NAD^+^ contents, HSYA improved Sirt1 activity to prevent HIF-1*α* accumulation, largely due to stabilizing VHL through deacetylation modification. These findings are consistent with the published study which demonstrated that Sirt1 induced VHL to promote HIF-1*α* degradation efficiently [17].

HIF-1*α* has been studied extensively during hypoxia. HIF-1*α* binds to the hypoxia-response elements to regulate gene induction involved in adaptive responses during ischemic injury [28]. However, accumulating evidence demonstrates the involvement of HIF-1*α*-associated inflammation in brain ischemic injury [29,30]. Although HIF-1*α* was able to increase core catalytic subunits of NOX1 and NOX2, we demonstrated that NOX2 was the predominant isoform in the cerebral microvascular endothelium, and the assembly of cytosolic regulatory subunits with membrane catalytic subunit may be the reason. It has been reported that, in human umbilical vein endothelial cells, NOX2 and HIF-1 were documented to mutually regulate each other to promote angiogenesis [31]. In the present study, we further revealed that HIF-1*α* induction of NOX2 in the context of inflammation and identified p47phox and Nox2 as target genes of HIF-1*α*. From the aspect of chemical structure, HSYA could donate reducing equivalents from the phenolic group to scavenge ROS, and its antioxidative effects have been well reported [11,25]; however, we addressed that targeting the enzymes that catalyze ROS generation should be a more important means to protect against ischemic injury.

TJ comprises a variety of transmembrane proteins, including claudin and occludin, as well as intracellular scaffold proteins such as ZO-1, -2, and -3 [5]. ZOs can bind to actin and vinculin-based cytoskeletal filaments, responsible for paracellular permeability in epithelia and endothelia. It was reported that proinflammatory cytokines reduced ZO-1 expression and occludin co-association in human brain microvascular endothelium [5]. In the present study, we found that ZO-1 protein stability was susceptible to oxidative stress. Accumulated ROS can produce a series of lipid peroxidation products such as 4-HNE and MDA, which can covalently modify proteins for oxidative degradation. The main pathway of protein degradation in mammals is through the proteasome system, including ubiquitin-dependent degradation by 26S proteasome and ubiquitin-independent proteolysis by 20S core proteasome [32]. The 26S proteasome comprises 20S peptidase and 19S regulatory particles, and actions through ATP-powered protein degradation machinery. Differently, oxidative damage causes protein degradation mainly through the 20S proteasome pathway regardless of ATP. Protein oxidation causes conformational changes, and exposure to hydrophobic residues is recognized by the 20S proteasome, promoting the opening of the *α*-rings and the degradation of the *β*-rings [20]. NQO1 could protect C/EBP*α* stability from 20S proteasomal degradation in protection against chemical-induced skin cancer [33]. We demonstrated that the 20S proteasome could bind to the oxidized ZO-1 protein to destroy its stability in the context of inflammation. When the 20S proteasome was knocked down, the protective effect of HSYA on ZO-1 protein was further potentiated. Given the specific impact of ZO-1 deficiency on TEER value and the diffusion of FITC–dextran, it is rational to believe that protecting ZO-1 stability against 20S proteasomal degradation by HSYA has a contribution to improving brain microvessel integrity from the aspect of redox homeostasis.

In mice subjected to a photothrombotic stroke, we also observed that HSYA protected cerebrovascular structure and function and reduced microglia aggregation at the infarct site. These results from the damaged brain provided evidence in vivo to support the findings observed in vitro. We should note that although HSYA stabilization of ZO-1 protected brain microvessel integrity, this was not the only way for its protection in vivo. Cerebral thrombosis is the main cause of stroke. Tissue plasminogen activator (t-PA) causes thrombus fibrinolysis, whereas plasminogen activator inhibitor-1 (PAI-1) leads to thrombus formation by inhibiting t-PA activity. HSYA was shown to improve the outcome following traumatic brain injury by enhancing the t-PA activity and decreasing the PAI-1 activity [34]. Together with brain microvessel integrity, alleviating neuronal apoptosis and microglial activation by HSYA should have a contribution to cerebral protection [13,25]. Furthermore, it has been reported that HSYA possesses vasodilation activity [35], implicating in antihypertension and pulmonary vascular remodeling [36]. Therefore, a comprehensive consideration focus on vascular characterization is necessary when we evaluate the protective role of HSYA during cerebral infarction.

In general, we conclude that HIF-1*α* induction of NOX2 activation underlies microvessel endothelial cell destruction during cerebral infarction, and protection of ZO-1 stability is an important strategy to improve cerebrovascular integrity. HSYA increased ZO-1 expression to rescue cerebrovascular endothelial cells from endotoxin insult by inhibiting HIF-1*α*/NOX2 signaling cascades, largely due to protecting redox homeostasis. This finding suggests the potential clinical application of HSYA for cerebrovascular protection.

## Data Availability

All of the data is contained within the article and the Supplementary Materials.

## Supplementary Materials

The following are available online at https://www.mdpi.com/article/10.3390/antiox11040728/s1, Figure S1: The structure of hydroxysafflor yellow A (HSYA); Figure S2: HSYA had no adverse effects on mice; Figure S3: HSYA suppressed inflammatory response; Figure S4: Negative and positive controls for intracellular cytoplasmic proteins and nucleoproteins; Figure S5: HSYA prevented VHL degradation; Figure S6: The expression of NOXs in bend.3 cells; Figure S7: Small interfering RNA (siRNA) screening; Figure S8: HSYA preserved ZO-1 protein stability; Table S1: Primer sequences for qRT-PCR; Table S2: Primer sequences for ChIP-qPCR.
