# Supplementary material for: Hydroxysafflor Yellow A Blocks HIF-1α Induction of NOX2 and Protects ZO-1 Protein in Cerebral Microvascular Endothelium
Source: Antioxidants (Basel). 2022 Apr 7;11(4):728. doi: 10.3390/antiox11040728 (PMC9025668; doi:10.3390/antiox11040728)
Supplement: Supplementary file 1 [file antioxidants-11-00728-s001.zip › antioxidants-1651848-supplementary.pdf]

Supplement Figures

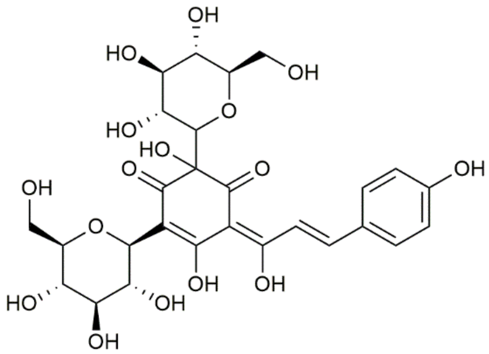

Figure S1. The structure of hydroxysafflor yellow A (HSYA).

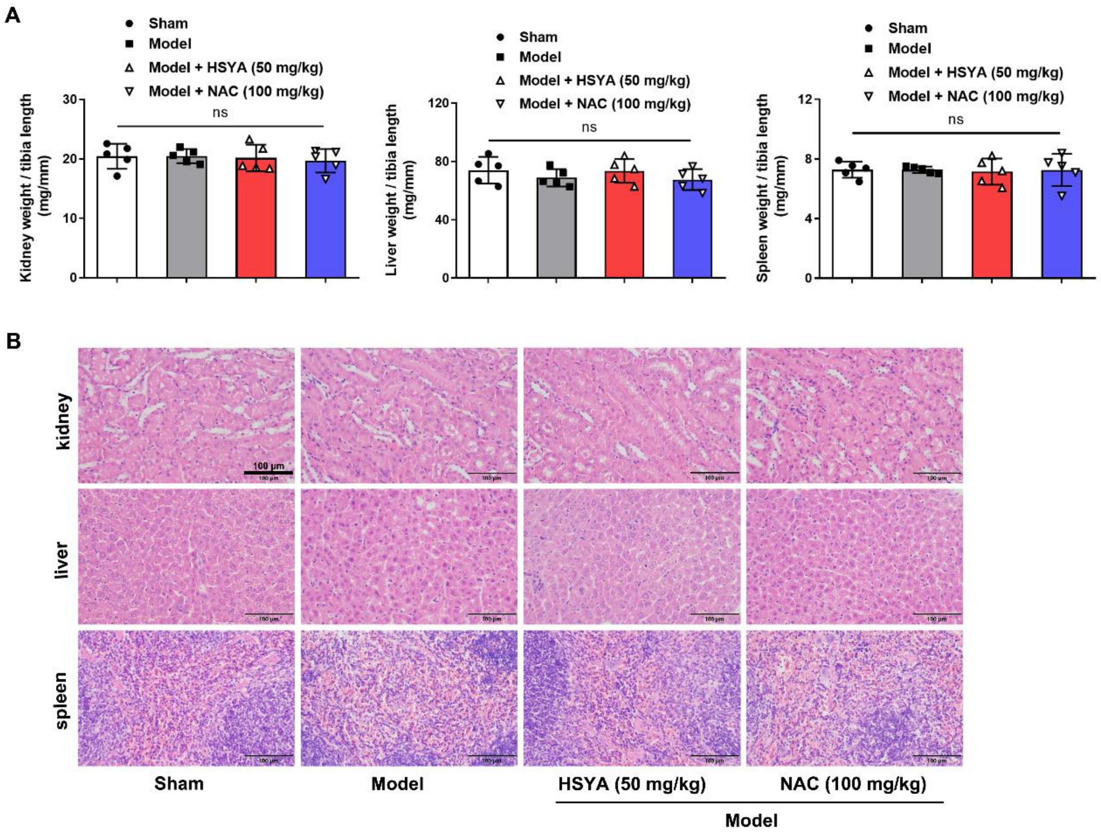

Figure S2. HSYA had no adverse effects on mice. Mice were intraperitoneally injected with HSYA and NAC for 3 days after photothrombotic stroke. **A** The kidney, liver and spleen of the mice were weighted and tibia length was measured. The ratios of kidney, liver and spleen mass to tibia length were calculated. **B** Histopathological examination in the kidney, liver and spleen tissue of the mice (scale bar: 100 µm). All data were presented as mean ± SD of five independent experiments. ns: no significant difference.

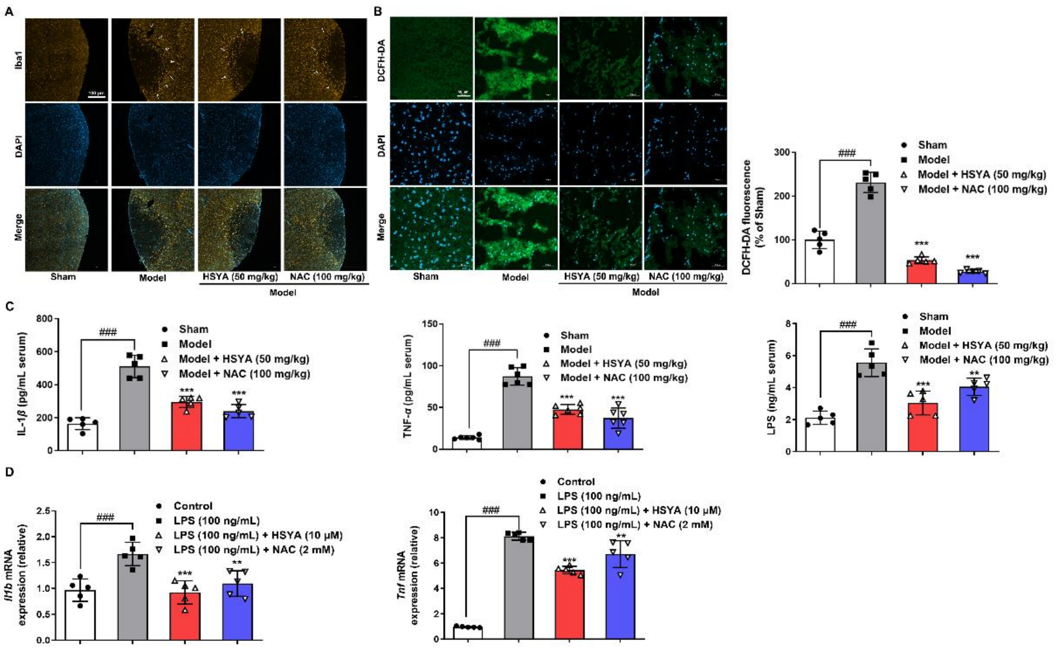

Figure S3. HSYA suppressed inflammatory response. Mice were intraperitoneally injected with HSYA and NAC for 3 days after photothrombotic stroke. **A** Immunofluorescence staining of Iba1 in the peri-infarct zones of brain tissue (scale bar: 100 μm). **B** ROS production was labeled with a DCFH-DA probe in the peri-infarct zones of brain tissue (scale bar: 50 μm). PL: peri-lesion. **C** The levels of IL-1*β*, TNF-*α*, and LPS in the serum were measured. **D** Gene expression of *Il1b* and tumor necrosis factor (*Tnf*) in bEnd.3 cells when exposed to LPS. All data were presented as mean ± SD of five independent experiments. $^{###}P < 0.001$ vs. indicated group, $^{**}P < 0.01$, $^{***}P < 0.001$ vs. LPS group.

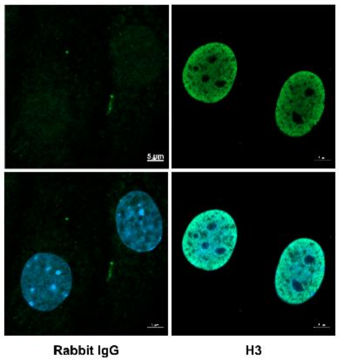

Figure S4. Negative and positive controls for intracellular cytoplasmic proteins and nucleoproteins. The expressions of IgG and histone 3 (H3) in bEnd.3 cells were observed by confocal laser microscopy (scale bar: 5 μm). All data were presented from five independent experiments.

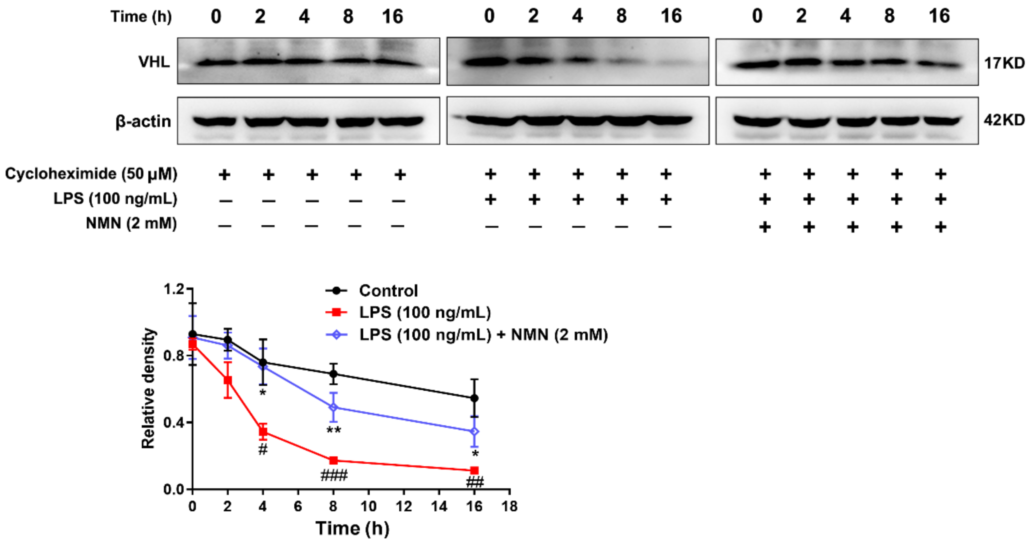

Figure S5. HSYA prevented VHL degradation. When protein synthesis was inhibited by cycloheximide in bEnd.3 cells, VHL protein degradation from 2 h to 16 h was determined by western blot. All data were presented as mean ± SD of five independent experiments. $^{\#}P < 0.05$, $^{\#\#}p < 0.01$, $^{\#\#\#}P < 0.001$ vs. Control group, $^{*}P < 0.05$, $^{**}P < 0.01$ vs. LPS group.

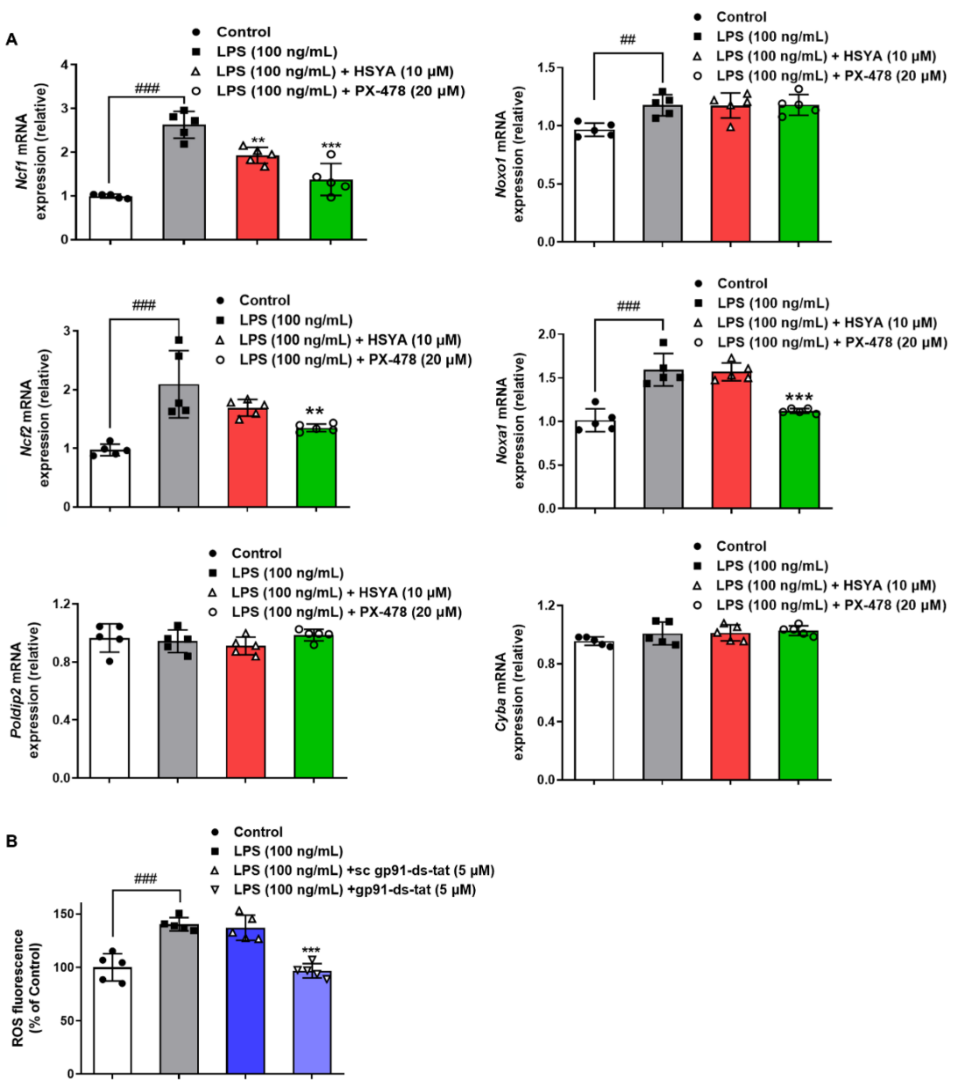

Figure S6. The expression of NOXs in bend.3 cells. A Gene levels of *Ncf1*, *Noxo1*, *Noxa1*, *Ncf2*, *Poldip2* and *Cyba* in LPS-induced bEnd.3 cells. **B** bEnd.3 cells were treated with sc gp91-ds-tat or gp91-ds-tat, and the intracellular ROS content was detected by fluorescein microplate analyzer. All data were presented as mean ± SD of five independent experiments. $^{\#\#}P < 0.01$, $^{\#\#\#}P < 0.001$ vs. Control group, $^{**}P < 0.01$, $^{***}P < 0.001$ vs. LPS group.

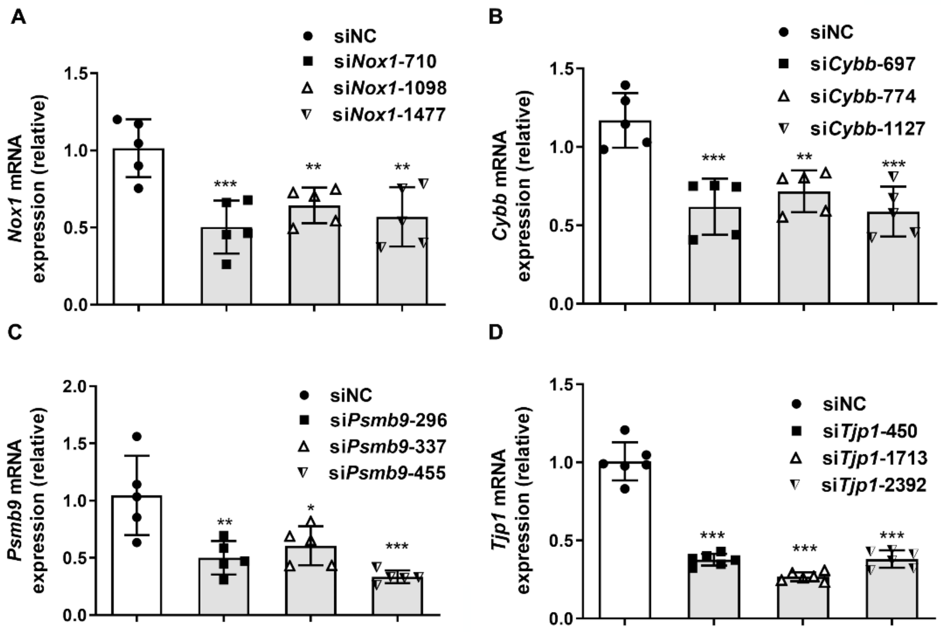

Figure S7. Small interfering RNA (siRNA) screening. bEnd.3 cells were transfected with si*Nox1*, si*Cybb*, si*Psmb9*, si*Tjp1* or siNC using lipofectamine 3000 transfection reagent for 48 h. **A-D** the gene levels of *Nox1*, *Cybb*, *Psmb9* and *Tjp1* were detected by qRT-PCR. All data were presented as mean ± SD of five or six independent experiments. **P* < 0.05, ***P* < 0.01, ****P* < 0.001 vs. siNC treatment.

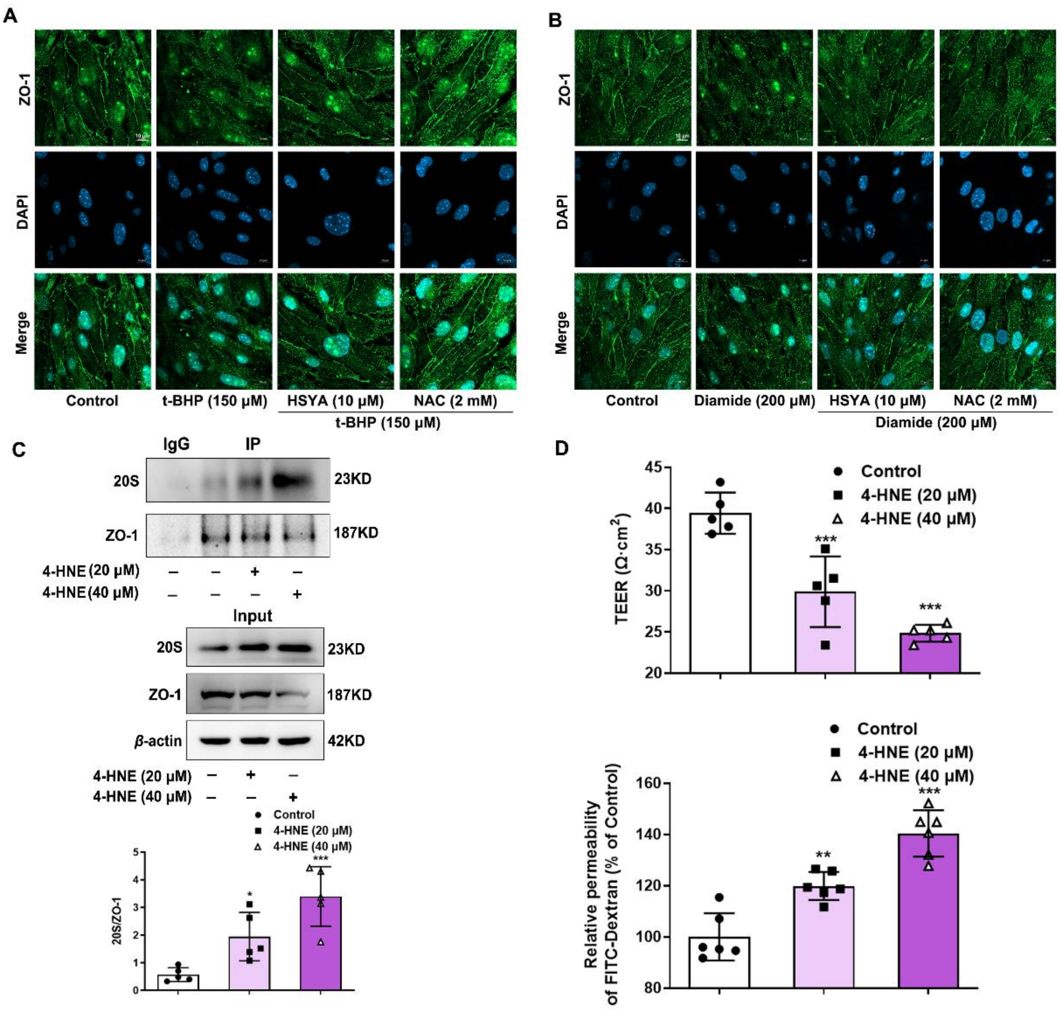

Figure S8. HSYA preserved ZO-1 protein stability. A-B Immunofluorescence observed ZO-1 protein expression in the presence of t-BHP or diamide (scale bar: 10 μm). **C** the binding of 20S to ZO-1 was determined by immunoprecipitation. **D** the TEER values and the absorbance of FITC-dextran across the bEnd.3 monolayer cells. All data were presented as mean ± SD of five independent experiments. $^{**}P < 0.01$, $^{***}P < 0.001$ vs. Control group.

Table S1. Primer sequences for qRT-PCR

Primers		Sequences	Gene accession number
Sod1	Forward	5'- GACTGCTGGAAAGGACGGTGTG -3'	AK020624.1
	Reverse	5'- ACGGCCAATGATGGAATGCTCTC -3'	
Cat	Forward	5'- CATAGCCAGAAGAGAAACCCACAGAC -3'	AK075853.1
	Reverse	5'- AATCCCTCGGTCACTGAACAAGAAAG -3'	
Ncf1	Forward	5'- TGGTGGGTGGTCAGGAAAGGG -3'	AK149668.1
	Reverse	5'- ATGCTCTGTGCGTTGCGGATG -3'	
Cybb	Forward	5'- CGAAGACAACTGGACAGGAACCTC -3'	AK053920.1
	Reverse	5'- AAATCCCGACTCTGGCATTCACAC -3'	
Tjp1	Forward	5'- GACCTTGAGCAGCCGTCATACAG -3'	AK144506.1
	Reverse	5'- CGTAGGCGATGGTCATAGTTCCG -3'	
Ocln	Forward	5'- TGGCTATGGAGGCGGCTATGG -3'	AK019880.1
	Reverse	5'- AAGGAAGCGATGAAGCAGAAGGC -3'	
Cldn 5	Forward	5'- TGGTGCTGTGTCTGGTAGGATGG -3'	AK077282.1
	Reverse	5'- GTCACGATGTTGTGGTCCAGGAAG -3'	
Il1b	Forward	5'- TCGCAGCAGCACATCAACAAGAG -3'	AK225002.1
	Reverse	5'- AGGTCCACGGGAAAGACACAGG -3'	
Tnf	Forward	5'- ATGTCTCAGCCTCTTCTCATTC -3'	AK153319.1
	Reverse	5'- GCTTGTCACTCGAATTTTGAGA -3'	
Psmb9	Forward	5'- GCGGGAACAGCAGTGGTGAAC -3'	AK008429.1
	Reverse	5'- CTTGGGCATCAGCAGCGGAAC -3'	
Nox1	Forward	5'- CCTGATTCCTGTGTGTCGAAA -3'	AK136432.1
	Reverse	5'- TTGGCTTCTTCTGTAGCGTTC -3'	
Nox4	Forward	5'- CCTTTTACCTATGTGCCGGAC -3'	AK050371.1
	Reverse	5'- CATGTGATGTGTAGAGTCTTGCT -3'	
Noxo1	Forward	5'- CTTGGTGCAAATGGACCGACT -3'	AF399754.1
	Reverse	5'- CCAGCTCCTCCGCACAAAT -3'	
Noxa1	Forward	5'- CTCTCTAGGGGATCAGATACGG -3'	AK087263.1
	Reverse	5'- GTCACTGCTTGGTCAAATGCC -3'	
Ncf2	Forward	5'- GGAGAAGTACGACCTTGCTATCA -3'	AK036379.1
	Reverse	5'- ACAGGCAAACAGCTTGAACTG -3'	
Poldip2	Forward	5'- TTTGAGGTGCCAAAACAAAATGG -3'	AK003702.1

	Reverse	5'- CCCTCGGTAGCCAAAAACAC -3'	
Cyba	Forward	5'- CTACTGCTGGACGTTTCACAC -3'	AK018713.1
	Reverse	5'- GGTGGACCCCTTTTTCCTCTT -3'	
Actb	Forward	5'- CTACCTCATGAAGATCCTGACC -3'	AK147787.1
	Reverse	5'- CACAGCTTCTCTTTGATGTCAC -3'	

Table S2. Primer sequences for ChIP-qPCR

**Primers**		**Sequences**	**Gene accession number**
Ncf1 promoter (site 1)	Forward	5'- TCCTCCTCCACTGCTGGAAT -3'	NC_000072
	Reverse	5'- CAGGAGGTCTCTGGGACTCA -3'	
Ncf1 promoter (site 2)	Forward	5'- ATCAGTCCCTGCTTTCTCCG -3'	
	Reverse	5'- AGATAGCCTGGCCTACGTGA -3'	
Cybb promoter (site 1)	Forward	5'- GCTGTCAGCATGGGTCTCTT -3'	NC_000086
	Reverse	5'- ACAGTTGTTGCCCTGGAGTC -3'	
Cybb promoter (site 2)	Forward	5'- AATGTGGGGCCTGAGGAATG -3'	
	Reverse	5'- TATCTTTCCGCACCTCCAGC -3'	
