# Supplementary figures and images for: Hydroxysafflor Yellow A Blocks HIF-1α Induction of NOX2 and Protects ZO-1 Protein in Cerebral Microvascular Endothelium
Source: Antioxidants (Basel). 2022 Apr 7;11(4):728. doi: 10.3390/antiox11040728 (PMC9025668; doi:10.3390/antiox11040728)

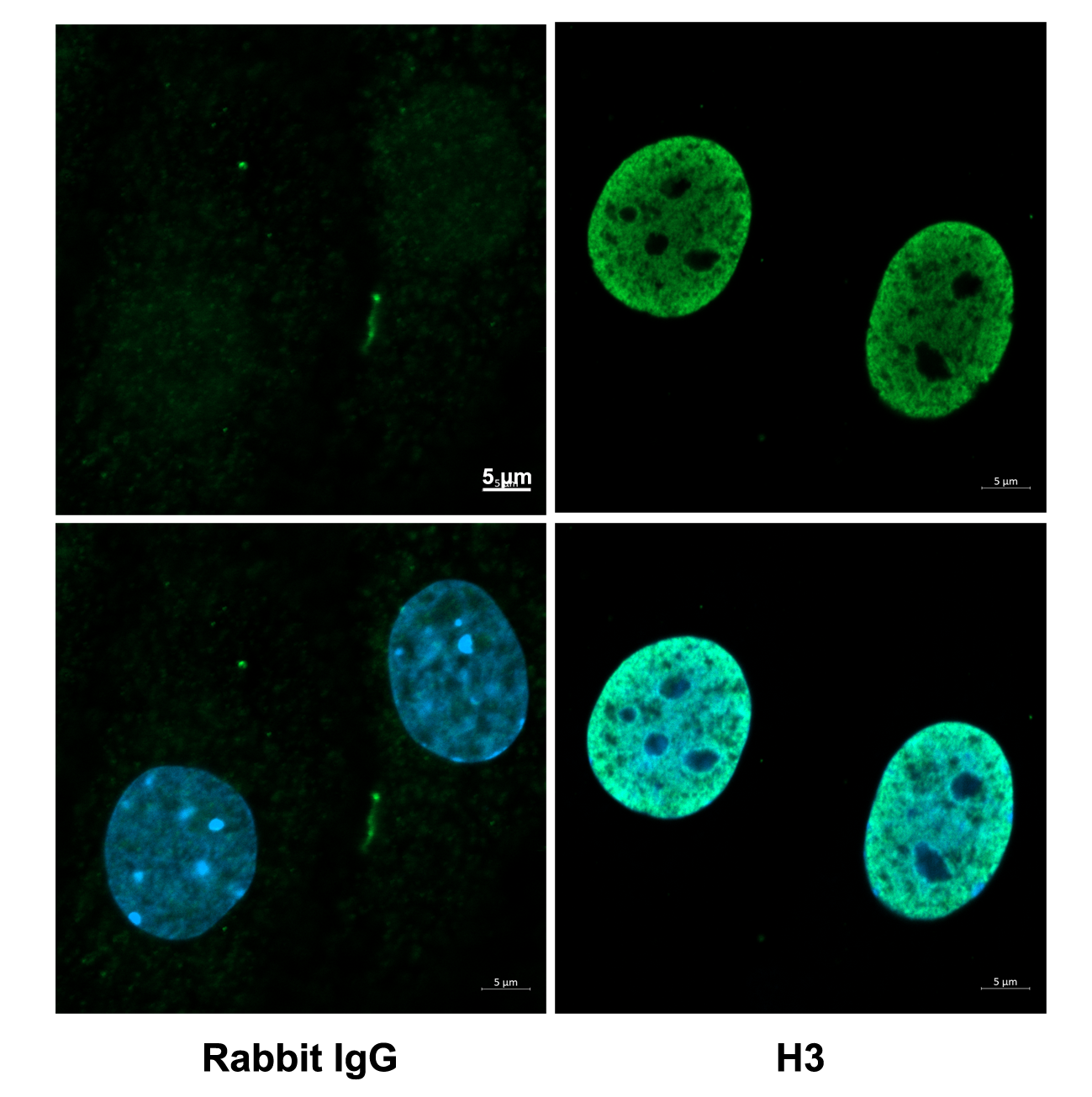

Supplement: Supplementary file 1 [file antioxidants-11-00728-s001.zip › Figure S4.tif]

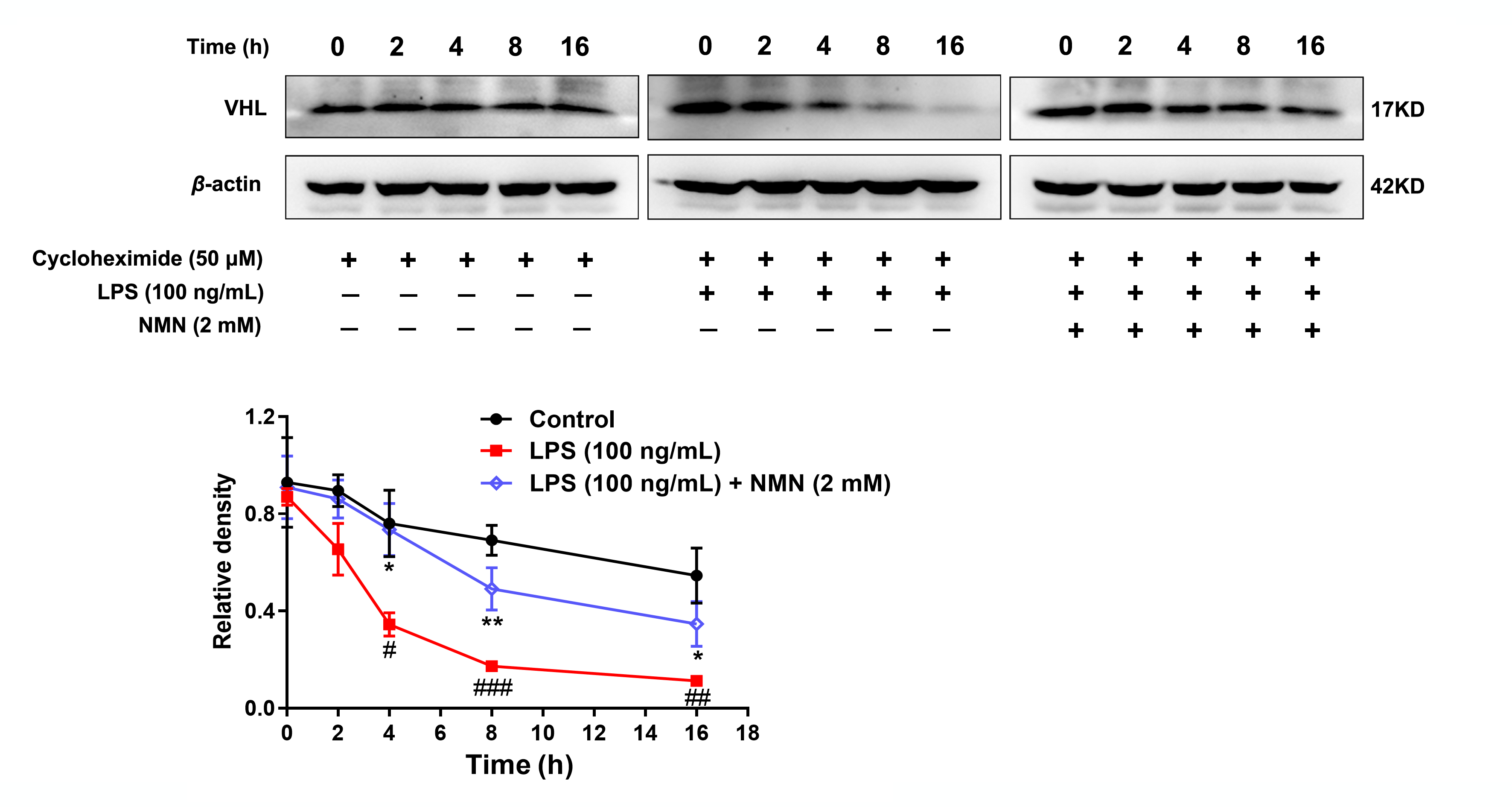

Supplement: Supplementary file 1 [file antioxidants-11-00728-s001.zip › Figure S5.tif]

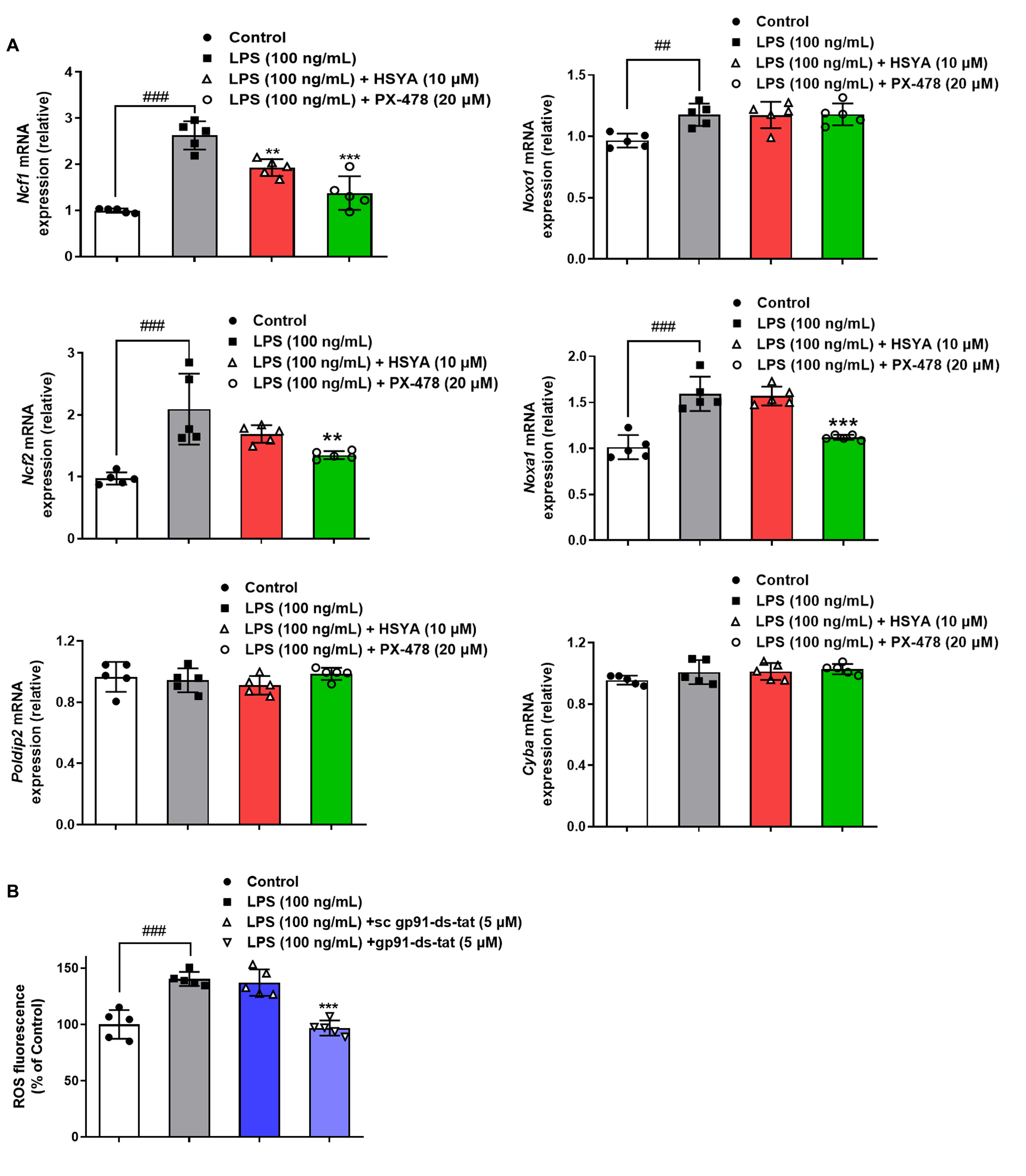

Supplement: Supplementary file 1 [file antioxidants-11-00728-s001.zip › Figure S6.tif]

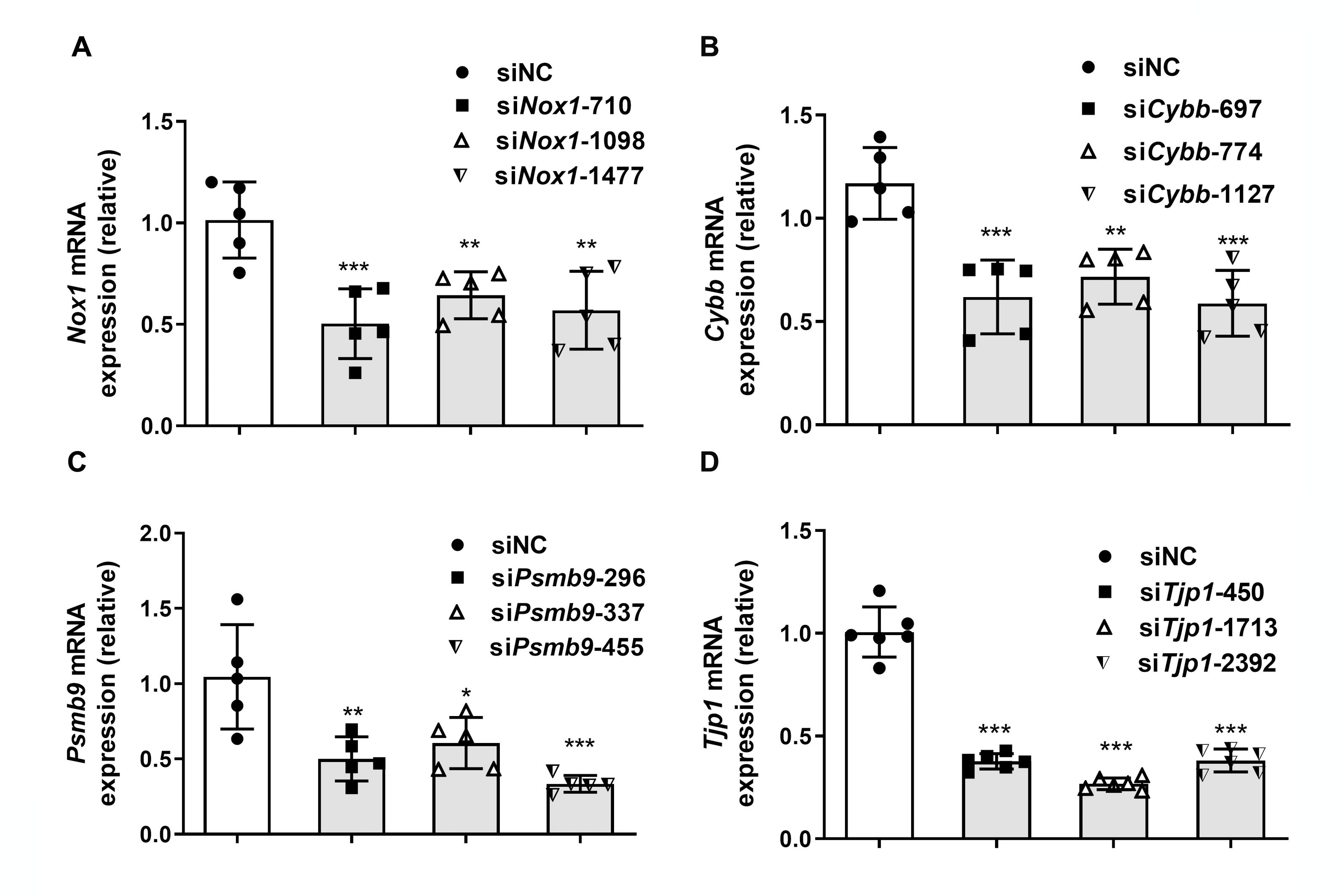

Supplement: Supplementary file 1 [file antioxidants-11-00728-s001.zip › Figure S7.tif]
